# The Effects of Continuous Compared to Accumulated Exercise on Health: A Meta-Analytic Review

**DOI:** 10.1007/s40279-019-01145-2

**Published:** 2019-07-02

**Authors:** Marie H. Murphy, Ian Lahart, Angela Carlin, Elaine Murtagh

**Affiliations:** 1 Centre for Exercise, Physical Activity, Medicine and Health, Ulster University, Jordanstown, Northern Ireland UK; 2 Faculty of Education, Health, and Wellbeing, University of Wolverhampton, Wolverhampton, England UK; 3 Department of Arts Education and Physical Education, Mary Immaculate College, University of Limerick, Limerick, Ireland

## Abstract

**Background:**

Public health guidelines suggest that physical activity can be accumulated in multiple short bouts dispersed through the day. A synthesis of the evidence for this approach is lacking.

**Objective:**

Our objective was to undertake a systematic review and meta-analysis to examine if exercise interventions consisting of a single bout of exercise compared with interventions comprising the same total duration, mode, and intensity of exercise accumulated over the course of the day have different effects on health outcomes in adults.

**Methods:**

Six electronic databases were searched (Jan 1970–29 August 2018). Two authors identified studies that evaluated the effects of a single bout of exercise compared with the same intensity, total duration, and mode of exercise accumulated in multiple bouts over the course of a day, in community-dwelling adults. Risk of bias was assessed using the Cochrane Collaboration tool. Pooled effects were reported as standardised mean differences (MDs) and 95% confidence intervals (CIs) using a random effects model.

**Results:**

A total of 19 studies involving 1080 participants met the inclusion criteria. There were no differences between accumulated and continuous groups for any cardiorespiratory fitness or blood pressure outcomes. A difference was found in body mass changes from baseline to post-intervention in favour of accumulated exercise compared with continuous (MD − 0.92 kg, 95% CI − 1.59 to − 0.25, *I*^2^ = 0%; five studies, 211 participants). In subgroup analyses, accumulating > 150 min of weekly exercise in multiple bouts per day resulted in small effects on body fat percentage (combined post-intervention and change from baseline values: MD − 0.87%, 95% CI − 1.71 to − 0.04, *I*^2^ = 0%; three studies, 166 participants) compared with 150 min of exercise amassed via single continuous bouts per day. There was a decrease in low-density lipoprotein (LDL) cholesterol with accumulated versus continuous exercise (MD − 0.39 mmol/l, 95% CI − 0.73 to − 0.06, *I*^2^ = 23%; two studies, 41 participants). No differences were observed for any other blood biomarker (total cholesterol, high-density lipoprotein cholesterol, triglycerides, fasting blood glucose, and fasting insulin).

**Conclusions:**

There is no difference between continuous and accumulated patterns of exercise in terms of effects on fitness, blood pressure, lipids, insulin and glucose. There is some evidence from a small number of studies that changes in body mass and LDL cholesterol are more favourable following the accumulated condition. Collectively our findings suggest that adults are likely to accrue similar health benefits from exercising in a single bout or accumulating activity from shorter bouts throughout the day. This review will inform public health guidelines for physical activity at the global and national levels (PROSPERO 2016 CRD42016044122).

**Electronic supplementary material:**

The online version of this article (10.1007/s40279-019-01145-2) contains supplementary material, which is available to authorized users.

## Key Points

**Table:** 

Splitting a continuous bout of exercise into shorter bouts of equivalent total duration spread over the course of a day does not alter its potential to provide health benefit.
For weight loss, splitting a single exercise bout into multiple bouts spread across the day may provide greater benefit.

## Introduction

Globally, approximately one quarter of adults (23.3%) are failing to meet current recommendations for physical activity [1]. Since 1995, the US physical activity guidelines have recommended that physical activity can be accumulated in shorter bouts across the day, totalling the recommended amount of physical activity for health [2]. Lack of time is frequently cited as a barrier to engagement in and adherence to physical activity [3, 4]. Framing the physical activity guidelines as shorter bouts that can be accumulated across the day as opposed to one continuous bout may present an easier means for individuals to achieve recommended levels of physical activity [5]. There is, however, limited research on the potential impact of prescribing shorter bouts [6]. Many physical activity guidelines have evolved to incorporate the recommendation that physical activity should be achieved in bouts of at least 10 min in duration [7, 8].

Acute responses to physical activity have been observed during and in the hours following a single bout of physical activity [9]. Reductions in ambulatory blood pressure [10], improvements in blood lipid profiles (through increases in high-density lipoprotein (HDL) cholesterol and reductions in triglyceride levels) and improved blood glucose control [11, 12] are all well-established acute responses to physical activity. Research has also identified the interaction that may exist between the acute and chronic responses to physical activity, highlighting that the repetition of acute, isolated sessions may eventually produce more permanent adaptations, similar to those observed in chronic exercise training studies [9].

Experimental findings have demonstrated that moderate-intensity physical activity, accumulated in shorter bouts (> 10 min in duration) and totalling at least 30 min in duration, may be as effective as longer bouts in improving certain disease risk factors, including lipid/lipoprotein profiles and fasting plasma insulin [13]. A previous review identified 16 primary studies comparing the health benefits of continuous versus accumulated physical activity interventions (i.e. exercise training interventions) [5]. The authors highlighted comparable benefits for cardiovascular fitness and normalisation of blood pressure between accumulated and continuous bouts of exercise [5]. A number of limitations of primary studies within the review were identified, including a lack of control group in studies (*n* = 7). Furthermore, the majority of included studies relied on self-reported measures of exercise (including the bouts frequency, intensity, and duration), which may have impacted the reliability of comparisons [5]. Integrating short bouts (modal duration of 10 min) of physical activity within organisational routines (e.g. schools, workplaces) has demonstrated modest increases in physical activity levels [14]. The variation in how bouts are prescribed within interventions and a lack of evidence on the measurement of bouts within such studies hinders conclusions regarding the minimum dose required for improvements in health-related markers [14].

Given that most free-living physical activity is accumulated in less structured periods of typically less than 10 min in duration (e.g. taking the stairs), the health benefits of shorter periods of activity warrant further investigation [15]. Undertaking sporadic physical activity (i.e. activities < 10 min duration) may also be associated with health benefits [16–18]. Cross-sectional evidence, involving the objective measurement of physical activity using accelerometers, has demonstrated that accumulating physical activity in shorter bouts (< 10 min) may favourably influence cardiometabolic risk factors, such as adiposity, blood lipids and glucose levels [17]. Moreover, accumulating physical activity—specifically at a moderate-to-vigorous intensity—in shorter bouts (< 10 min) may present a feasible option for individuals wishing to increase their physical activity and lower subsequent disease risk [18]. Contrary to the above, cross-sectional evidence has also highlighted that longer bouts undertaken for greater than 10 min may be more predictive of lower levels of obesity markers [18]. This may be attributed to the higher relative intensity of the types of physical activity that is undertaken in bouts longer than 10 min [18].

More recently, research has sought to investigate the effects of very short bouts (< 1 min) of very vigorous, near maximal intensity exercise—specifically high-intensity interval training (HIIT)—on health, but these studies have been reviewed elsewhere [19] and are not included in this review. The aim of this systematic review was to investigate the effects of chronic exercise training interventions (i.e. ≥ 4 weeks duration) consisting of single bouts of moderate-to-vigorous exercise performed per day (continuous exercise) compared with interventions comprising exercise of the same total duration and intensity accumulated over the course of a day (accumulated exercise) on health-related outcomes and exercise adherence.

Accordingly, a meta-analysis was undertaken to synthesise the effects of continuous versus accumulated bouts on exercise levels, cardiorespiratory fitness, resting cardiovascular outcomes, anthropometric and body composition outcomes, blood biomarkers, and psychological outcomes.

## Methods

The protocol for this review was pre-registered on PROSPERO (https://www.crd.york.ac.uk/prospero/display_record.php?RecordID=44122).

### Search Strategy

We searched the following electronic databases: PubMed, EMBASE, the Cochrane Central Register of Controlled Trials (CENTRAL), Cumulative Index to Nursing and Allied Health Literature (CINAHL), PEDro, and SPORTDiscus. Keywords used for the search included the following: single bout, multiple bout, short bout, long bout, intermittent, continuous, accumulate, exercise, physical activity, walk, sport, resistance, running, cycling, and swimming (see Electronic Supplementary Material Appendix S1 for search strategies). Databases were initially searched from inception to June 2017, and an updated search was conducted on 29 August 2018.

### Trial Selection

Titles and abstracts of potentially eligible trials were screened independently by two review authors (AC and MM). The full texts of all trials that were not excluded after initial title and abstract screening were retrieved and independently assessed for eligibility by two authors (AC and EM). Disagreements between researchers during full-text screening were resolved through discussion with a third reviewer (MM). We collated multiple publications for the same eligible trials and used the most recent or complete report (i.e. the one with outcomes most relevant to the review) as the primary reference.

A trial was considered eligible if it met the inclusion criteria provided in Table 1. In brief, we included any trials that evaluated the longitudinal effects of single daily bouts of exercise (continuous) compared with the same daily and weekly dose of exercise—performed at the same intensity and using the same mode—accumulated in multiple bouts over the course of a day (accumulated).

**Table 1:** Eligibility criteria

Population	Inclusion: free-living, community-dwelling adults (age ≥ 18 years) Exclusion: children and adolescents (< 18 years), people living in residential care
Intervention	Inclusion: exercise training interventions of at least 4 weeks in duration comprising accumulated exercise in multiple bouts over the course of a day Exclusion: intermittent exercise performed in the same exercise session (e.g. high-intensity intermittent exercise, characterised by repeated short bouts of high-intensity exercise separated by brief periods of low-intensity activity or rest)
Comparisons	Inclusion: (1) Exercise training interventions comprising single bouts of exercise per day at the same intensity, mode, and total daily, weekly, and intervention duration as the accumulated exercise in multiple bouts conditions (2) No exercise control group observed for the same total intervention duration Exclusion: studies that compared different modes and intensities of exercise, as well as studies that compared different total daily, weekly, and intervention durations of exercise
Outcomes	We did not exclude on the basis of outcomes Based on our previous review [5], we expected health outcomes to include anthropometric (e.g. body mass, body fat, hip and waist circumference), physiological (e.g. cardiovascular fitness), biochemical (e.g. blood biomarkers) and psychological/psychosocial (e.g. quality of life, self-esteem, motivation, mood, self-efficacy)
Study design	Inclusion: longitudinal randomised, quasi-randomised, or non-randomised comparative trials, and randomised, quasi-randomised, or non-randomised controlled trials Exclusion: Single group, cohort, and cross-sectional trials, and trials investigating the acute effects of exercise
Other limits	Full publications in the English language

### Outcomes

Our primary outcomes included (1) cardiorespiratory fitness [e.g. maximal oxygen uptake (*V*O_2max_)]; (2) body fatness (e.g. body fat percentage); and (3) cardiovascular risk factors (e.g. blood pressure and blood lipids) measured using standard techniques. Secondary outcomes included psychological/psychosocial parameters (e.g. quality of life, self-esteem, motivation, mood, and self-efficacy), other anthropometric measures (e.g. lean mass and waist-to-hip ratio), and objectively measured physical activity and sedentary behaviour derived from accelerometers. We also recorded adverse events and adherence to exercise programmes.

### Data Extraction

Two authors (AC, IL) independently extracted data from eligible trials, and EM and MM arbitrated any conflicts not due to extractor error. Data extraction included, in addition to outcomes, information regarding study design, country of origin, number of participants included in each condition, participants characteristics (including age, sex, mass, body mass index (BMI), baseline physical activity, and ethnicity), intervention characteristics (including dose, mode, setting, frequency, intensity, and duration of exercise, number of bouts per day, time between bouts, progression in exercise frequency, intensity, and duration), details of control groups, intervention adherence, measurement timings, trial attrition, and information for assessment of risk of bias. For each outcome, we recorded the definition, unit of measurement and scales, assessment time points, results including numbers of participants analysed, missing data with reasons, summary of data for each group (mean post-intervention values with corresponding standard deviation (SD), and mean change from baseline to post-intervention scores with SD), and effect estimates with confidence intervals (CIs), if provided. Relevant data provided only in figures were extracted using WebPlotDigitizer 4.1 software (https://automeris.io/WebPlotDigitizer).

### Risk of Bias Assessment

The assessment of risk of bias in trials was assessed using the Cochrane Collaboration assessment tool [20]. We made judgements regarding the level of risk (low, high, or uncertain) for selection bias (allocation sequence generation and allocation concealment), performance bias (blinding of participants and personnel), detection bias (blinding of outcome assessors), attrition bias (incomplete outcome data), selective outcome reporting bias, and other bias (baseline imbalances and exercise adherence), according to the Cochrane Handbook for Systematic Reviews of Interventions. If trials did not mention that outcome assessors were blinded to participant allocation, we assumed that they were not blinded, and judged these trials at a high risk of detection bias. We considered trials with > 20% of data missing as having a high risk of attrition bias. Similarly, trials with baseline imbalances or less than 75% adherence in the intervention group were judged to be at a high risk of other bias.

Trials with a low risk of bias for all of the biases above—except for performance bias (it is not possible to blind participants to an exercise intervention)—were considered ‘trials at low risk of bias’. Trials assessed as having uncertain or high risk of bias in two or more of the above specified domains (except performance bias) were considered trials at ‘high risk of bias’. Trials at low risk of bias in the allocation concealment, blinded outcome assessment, and the incomplete outcome data domains were characterised as ‘trials potentially having less high risk of bias’ compared with other trials at high risk of bias [21].

### Data Synthesis and Analysis

Where data were available from two or more trials, we performed a meta-analysis. For outcomes where insufficient data were available to pool, we presented the results qualitatively. All outcome data included were continuous in nature.

In accordance with the Cochrane Handbook’s recommendations [22], we utilised the inverse variance random-effects method for all meta-analyses to combine data [23]. All analyses were conducted using Review Manager 5 (version 5.3) [24]. Mean ± SD data for either change from baseline to post-intervention (change scores) or post-intervention values were combined in a meta-analysis. The RevMan calculator was used to convert standard errors, CIs, or *t* values to SD where necessary. We have presented pooled intervention effect estimates and their 95% CI. Mean difference (MD) data were presented when all trials reported the same outcome using the same scale. If this was not possible, standardised mean difference (SMD) was used. SMD is the mean difference in scores between the accumulated and continuous exercise groups divided by the pooled SD at follow-up. By convention, SMD effect sizes of 0.2, 0.5, and 0.8 are considered small, medium, and large intervention effects, respectively.

In trials that contained more than one eligible intervention arm, outcome data from both groups were combined using methods recommended by Deeks et al. [22]. Where included trials used a crossover design, then only data up to the point of crossover were used.

We evaluated inconsistency of results across studies by using the *I*^2^ statistic, which provides the proportion of variation observed between the trials attributable to between-trial differences versus sampling error (i.e. chance). Consistent with Higgins et al. [20] (i.e. Cochrane Handbook), we interpreted *I*^2^ values of 0–40% as ‘might not be important’, 30–60% as ‘may represent moderate heterogeneity’, 50–90% as ‘may represent substantial heterogeneity’, and 75–100% as showing ‘considerable heterogeneity’. However, the importance attached to the observed value of *I*^2^ depends on the magnitude and direction of effects and the strength of evidence of heterogeneity (e.g. *p* value from the Chi^2^ test, CI for *I*^2^). When we found evidence of at least substantial heterogeneity, its potential source was investigated by (1) removal of the largest outlier from the analysis, (2) comparing different exercise doses, and (3) comparing trials at low versus high risk of bias.

Subgroup analysis by exercise dose (< 150 min/week vs. 150 min/week vs. > 150 min/week) was conducted where there were data from two or more studies. Similarly, when there were a sufficient number of trials available, we planned to conduct sensitivity analyses to assess the robustness of results by removing studies with high or unclear risk of bias. We investigated publication bias using funnel plots to explore the possibility of small study effects (i.e. a tendency for smaller studies to report larger beneficial effects), but only if there were at least ten trials included in an analysis.

## Results

**Fig. 1:**
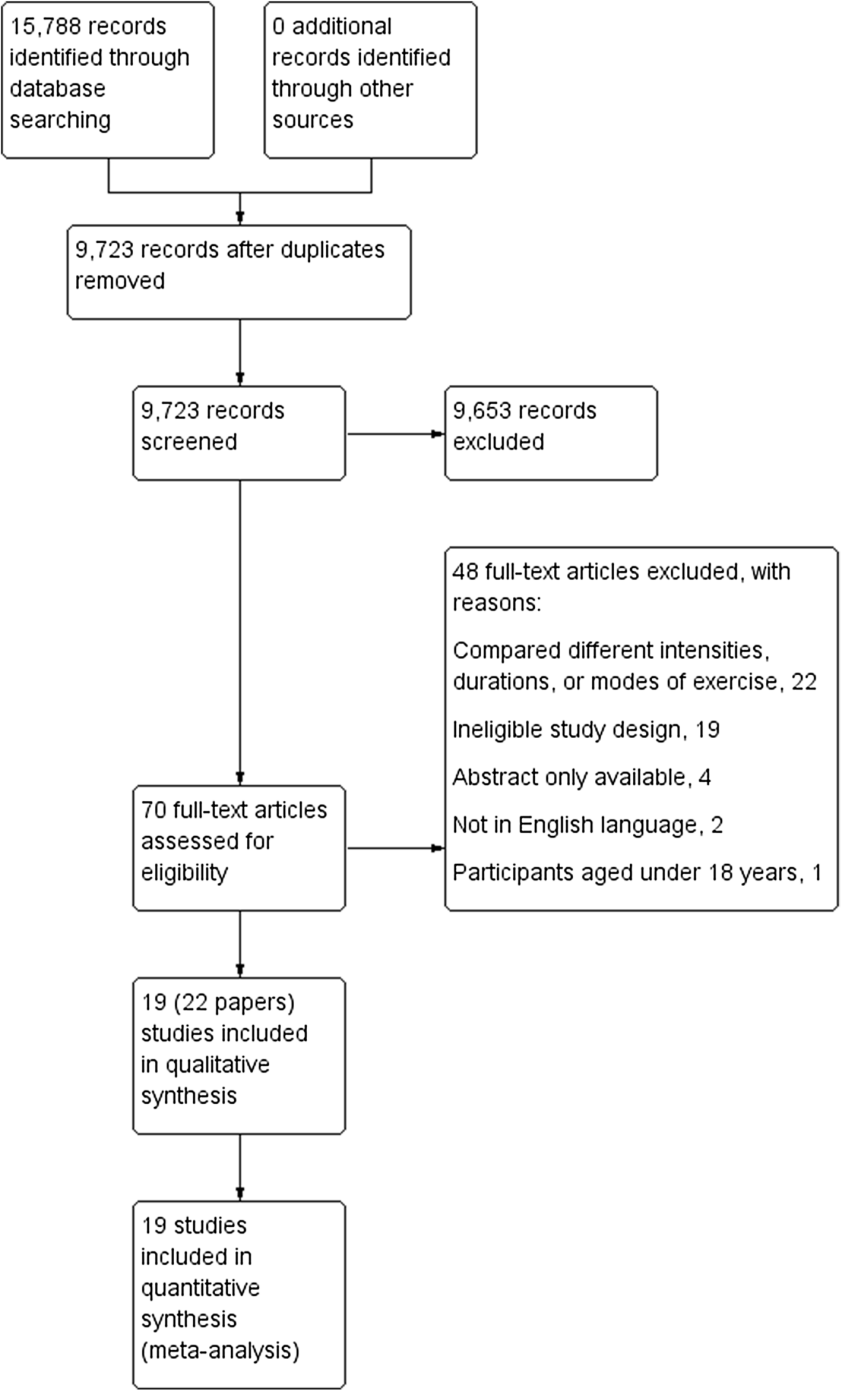
Study flow diagram

A total of 9723 article titles and abstracts were reviewed. From this process we identified 70 full-text articles to review, and of these, 22 articles based on 19 trials were eligible (see Fig. 1). Below we provide a summary of the key characteristics (participants, intervention, exercise adherence, and outcome details) of these eligible trials (see Table 2 for an overview of characteristics per trial).

**Table 2:** Study characteristics

Study, country	No. randomised/analysed	Participants	Exercise prescription and control condition	Adherence in exercise groups	Outcomes assessed
Alizadeh et al. [26–28], Iran	Total: 45/31 Accumulated: 15/10 Continuous: 15/9 Control: 15/12	Description: sedentary, overweight/obese. Sex: 100% F. Mean (SD) age 33.3 ± 7.8 years. Mean (SD) BMI: 30.6 ± 4.1 kg/m^2^. Mean (SD) BF%: 37.7 ± 3.6%	Intervention duration: 12 weeks Mode: walking Intensity: 64–76% APHR max Setting: home-based Format: individual, unsupervised Progression: yes, met target exercise duration by week 3 Accumulated condition (days/week × bouts/day × min): 5 days × 3 × 13.3 min Continuous condition (days/week × bouts/day × min): 5 days × 1 × 40 min Control condition: continue with usual physical activity All trial participants consumed 500 kcal less than their Harris Benedict equation-derived daily energy intake	% of sessions completed: Accumulated: 66% Continuous: 96% Mean (SD) min/day of walking: Accumulated: 28.5 (11.4) min/day Continuous: 34.2 (3.7) min/day	Anthropometrics: mass, BMI, WC, SKF, BIA Physical activity: log book Other: self-reported energy intake
Altena et al. [32], USA	Total: 18/18 Accumulated: 8/8 Continuous: 10/10	Description: untrained, non-obese normo-lipidaemic individuals. Sex: 61.1% F. Mean (SD) age: 25.0 ± 1.8 years. Mean (SD) BMI: 23.2 ± 0.8 kg/m^2^. Mean (SD) BF%: 21.2 ± 1.9%	Intervention duration: 4 weeks Mode: jogging (treadmill) Intensity: 60% *V*O_2max_ or 75% HR_max_ Setting: exercise laboratory setting Format: individual, unsupervised Progression: no Accumulated condition: 5 days × 3 × 10 min Continuous condition: 5 days × 1 × 30 min Control condition: no control	NR	Anthropometrics: mass, BMI, WC, HC, WHR, SKF Blood biomarkers: total cholesterol, LDL-C, HDL-C, triglycerides CR fitness: *V*O_2max_; RPE Other: dietary habits, caloric expenditure
Asikainen et al. [25, 29, 30], Finland	Total: 134/130 Accumulated: 43/43 Continuous: 46/44 Control: 45/43	Description: healthy, non-obese, sedentary, postmenopausal women. Sex: 100% F. Mean (SD) age: 57.3 ± 4.3 years. Mean (SD) BMI: 25.8 ± 2.8 kg/m^2^. Mean (SD) BF%: 37.1 ± 4.6%	Intervention duration: 15 weeks Mode: walking Intensity: 65% *V*O_2max_ Setting: indoor track and home-based Format: individual, supervised/unsupervised Progression: yes, target exercise duration met by week 2 Accumulated condition: 5 days × 2 × 150 kcal (mean: 24 min/bout) Continuous condition: 5 days × 1 × 300 kcal (mean: 47 min/bout) Control condition: attended monthly meeting with lectures on health topics and a few minutes of light flexibility exercises	% of prescribed sessions completed: Accumulated: 95% Continuous: 89%	Anthropometrics: mass, BMI, SKF Blood biomarkers: total cholesterol, LDL-C, HDL-C, triglycerides, blood glucose, plasma insulin CV outcomes: SBP, DBP CR fitness: *V*O_2max_; Health-related Fitness Test Battery Physical activity: self-report diary and habits, pedometer Other: exercise-related pain and injuries
Chung et al. [40], South Korea	Total: 47/36 Accumulated: 17/12 Continuous: 16/12 Control: 14/12	Description: middle-aged obese women. Sex: 100% F. Mean age: 49 years. Mean BMI: 25.1 kg/m^2^. Mean BF%: 35.9%	Intervention duration: 12 weeks Mode: walking (treadmill) Intensity: moderate (average 83% *V*O_2max_; 200 kcal in 30 min) Setting: public health centre Format: individual, supervised Progression: yes, target exercise duration met by week 7 Accumulated condition 1: 3 days × 3 × 10 min Continuous condition: 3 days × 1 × 30 min Control condition: no exercise	NR	Anthropometrics: mass, BMI, FM, BF%, FFM via BIA, and waist circumference Blood biomarkers: total cholesterol, LDL-C, HDL-C, triglycerides, atherogenic index, blood glucose CV outcomes: SBP, DBP
Coleman et al. [41], USA	Total: 36/32 Accumulated 1: 11/11 Accumulated 2: 11/11 Continuous: 10/10	Description: sedentary university employees. Sex: 84% F. Mean (SD) age: 39.9 ± 8.7 years. Mean (SD) BMI: 25.8 ± 4.0 kg/m^2^. Mean (SD) BF%: 32.3 ± 2.4%	Intervention duration: 16 weeks Mode: walking Intensity: moderate Setting: home-based Format: individual, unsupervised Progression: yes, target exercise duration met by week 7 Accumulated condition 1: 6 days × 3 × 10 min Accumulated condition 2: 6 days × as many bouts of ≥ 5 min duration totalling 30 min Continuous condition: 6 days × 1 × 30 min Control condition: no control	% of weekly meetings attended: Accumulated 1: 96.6% Accumulated 2: 95.5% Continuous: 93.8% Mean (SD) objectively measured minutes walking ≥ 3.0 METs: Accumulated 1: 38 (23) min, week 16 Accumulated 2: 37 (21) min, week 16 Continuous: 36 (17) min, week 16	Anthropometrics: mass, BMI, BIA CV outcomes: SBP, DBP CR fitness: *V*O_2max_ Physical activity: self-report diary, accelerometer Other: Binge Eating Scale, Symptoms Checklist-90, feedback questionnaires
DeBusk et al. [45], USA	Total: 40/36 Accumulated: 20/18 Continuous: 20/18	Description: sedentary healthy male adults. Sex: 0% F. Mean (SD) age: 51.5 ± 6.0 years	Intervention duration: 20 weeks Mode: jogging Intensity: 65–75% peak HR Setting: home/work Format: individual, unsupervised Progressive: no Accumulated condition: 5 days × 3 × 10 min Continuous condition: 5 days × 1 × 30 min Control condition: no control	% of sessions completed: Accumulated: 92% Continuous: 93%	Anthropometrics: mass CV outcomes: SBP, DBP CR fitness: *V*O_2max_; HR and RPE (self-report) Physical activity: self-report exercise log Other: level of enjoyment, level of convenience
Eguchi et al. [46], Japan	Total: 23/23 Accumulated: 12/12 Continuous: 11/11	Description: sedentary male workers. Sex: 0% F. Mean (SD) age: 43.9 ± 11.6 years. Mean (SD) BMI: 25.8 ± 3.2 kg/m^2^	Intervention duration: 20 weeks Mode: cycling Intensity: 50% *V*O_2max_ Setting: near workplace Format: individual, unsupervised Progressive: no Accumulated condition: 3 days × 3 × 10 min Continuous condition: 3 days × 1 × 30 min Control condition: no control	% of prescribed minutes completed: Accumulated: 55.5% Continuous: 68.9%	Anthropometrics: mass, BMI, WC Blood biomarkers: total cholesterol, LDL-C, HDL-C, triglycerides, FPI, FPG, TBARS, and HOMA-IR CV outcomes: resting SBP, resting DBP CR fitness: resting HR, *V*O_2max_
Jakicic et al. [44], USA	Total: 56/52 Accumulated: 28/25 Continuous: 28/27	Description: overweight sedentary female adults. Sex: 100% F. Mean (SD) age: 40.7 ± 6.6 years. Mean (SD) BMI: 33.9 ± 4.1 kg/m^2^	Intervention duration: 20 weeks Mode: mainly walking Intensity: 70% HRR Setting: home-based Format: individual, unsupervised Progressive: yes, target exercise duration met by week 9 Accumulated condition: 5 days × 4 × 10 min Continuous condition: 5 days × 1 × 40 min Control condition: no control Recommended caloric intake for all subjects was 5022–6277 kJ/day (1200–1500 kcal), with fat reduced to 20% of caloric intake	% of prescribed minutes completed: Accumulated: 110% weeks 1–4; 67% weeks 17–20 Continuous: 112% weeks 1–4; 91% weeks 17–20	Anthropometrics: mass, BMI, CV outcomes: resting SBP, resting DBP CR fitness: resting HR, *V*O_2max_ Physical activity: self-report exercise records, accelerometer) Other: caloric expenditure, dietary intake
Jakicic et al. [42], USA	Total: 148/115 Accumulated 1: 51/36 Accumulated 2: 48/42 Continuous: 49/37	Description: overweight sedentary adults. Sex: 100% F. Mean (SD) age: 36.7 ± 5.6 years. Mean (SD) BMI: 32.8 ± 4.0 kg/m^2^. Mean (SD) BF%: 44.3 ± 4.7%	Intervention duration: 72 weeks Mode: brisk walking Intensity: NR Setting: home-based Format: individual, unsupervised Progressive: yes, target exercise duration reached by week 9 Accumulated condition 1: 5 days × 4 × 10 min (no home treadmill) Accumulated condition 2: 5 days × 4 × 10 min (provided with home treadmill) Continuous condition: 5 days × 1 × 40 min Control condition: no control Instructed to reduce daily energy intake and fat intake. Subjects weighing ≥ 90 kg at baseline prescribed an intake of 6276 kJ/day; subjects < 90 kg prescribed 5021 kJ/day	Adherence (% of sessions attended): Accumulated 1: 70.9% Accumulated 2: 71.7% Continuous: 67.1%	Anthropometrics: mass, BMI, WC, HC, WHR, BF% CR fitness: *V*O_2max_ Physical activity: self-report weekly exercise records, leisure time physical activity questionnaire, accelerometer) Other: dietary intake
Murphy and Hardman [35], Northern Ireland	Total: 47/34 Accumulated: 16/12 Continuous: 16/12 Control: 15/10	Description: previously sedentary individuals. Sex: 100% F. Mean (SD) age: 46.7 ± 6.0 years. Mean (SD) BMI: 25.8 ± 3.6 kg/m^2^	Intervention duration: 10 weeks Mode: brisk walking Intensity: 70–80% of HR_max_ Setting: outdoor, university campus Format: individual, unsupervised with 1 day/week supervised Progressive: no Accumulated condition: 5 days × 3 × 10 min Continuous condition: 5 days × 1 × 30 min Control condition: no training	% of prescribed sessions completed: Accumulated: 85% Continuous: 88%	Anthropometrics: mass, WC, SKF CR outcomes: resting SBP CR fitness: *V*O_2max_ Physical activity: training diary
Murphy et al. [33], Northern Ireland	Total: 32/21 Accumulated: 19/13 Continuous: 11/8	Description: normo-lipidaemic sedentary adults. Sex: 67% F. Mean (SD) age: 44.5 ± 6.1 years. Mean (SD) BMI: 26.8 ± 3.5 kg/m^2^	Intervention duration: 6 weeks Mode: walking Intensity: 70–80% predicted HR_max_ Setting: outdoors, home-based Format: individual, unsupervised Progressive: no Accumulated condition: 5 days × 3 × 10 min Continuous condition: 5 days × 1 × 30 min Control condition: no control	% of prescribed sessions completed: Accumulated: 88.2% Continuous: 91.3%	Anthropometrics: mass, WC, HC, SKF Blood biomarkers: total cholesterol, HDL-C, triglycerides CV outcomes: resting SBP, resting DBP CR fitness: *V*O_2max_ Physical activity: training diary Other: mood, barriers to exercise scale, self-efficacy
Murtagh et al. [36], Northern Ireland	Total: 48/32 Accumulated: 18/9 Continuous: 19/15 Control: 11/8	Description: Healthy, inactive university staff. Sex: 65% F. Mean (SD) age: 45.7 ± 9.4 years. Mean (SD) BF%: 27.8 ± 6.9%	Intervention duration: 12 weeks Mode: walking (treadmill) Intensity: brisk Setting: university gym Format: individual, unsupervised, 1 supervised session/week Progressive: no Accumulated condition: 3 days × 2 × 10 min Continuous condition: 3 days × 1 × 20 min Control condition: no training	% of sessions completed: Accumulated: 82.1% Continuous: 90.4%	Anthropometrics: mass, BMI, WC, HC, BIA Blood biomarkers: total cholesterol, LDL-C, HDL-C, triglycerides, haemoglobin, haematocrit CV outcomes: SBP, DBP CR fitness: *V*O_2max_; HR and RPE (self-report) Physical activity: self-report
Osei-Tutu and Campagna [37], Canada	Total: 40/30 Accumulated: 15/9 Continuous: 15/11 Control: 10/10	Description: Healthy, sedentary. Sex: 47.5% F. Mean (SD) age: 34.0 ± 5.5 years. Mean (SD) BF%: 26.2 ± 1.3%	Intervention duration: 8 weeks Mode: walking Intensity: 60–79% of HR_max_ Setting: home-based Format: individual, unsupervised Progressive: yes, target exercise duration met by week 3 Accumulated condition: 5 days × 3 × 10 min Continuous condition: 5 days × 1 × 30 min Control condition: remain sedentary and make no lifestyle changes. Met with the researcher after the 2nd, 4th and 6th week of experiment to discuss fitness goals and provide input to the type of exercise training programme desired	NR	Anthropometrics: SKF CR fitness: *V*O_2max_ Physical activity: log book Mood: profile of mood states, total mood disturbance
Quinn et al. [34], USA	Total: 45/37 Accumulated: 23/20 Continuous: 22/17	Description: moderately active individuals. Sex: 54.1% F. Mean (SD) age: 49.1 ± 8.7 years. Mean (SD) BF%: 28.9 ± 6.2%	Intervention duration: 12 weeks Mode: aerobic (variety of modalities) Intensity: final intensity 70–80% HRR Setting: home-based Format: individual, unsupervised Progressive: yes, target exercise intensity met by week 3 Accumulated condition: 4 days × 2 × 15 min Continuous condition: 4 days × 1 × 30 min Control condition: no control	% of prescribed exercise time completed: Accumulated: 96.30% Continuous: 96.60%	Anthropometrics: WC, HC, WHR, SKF Blood biomarkers: total cholesterol, LDL-C, HDL-C, triglycerides, total cholesterol:HDL-C ratio CV outcomes: SBP, DBP during exercise CR fitness: *V*O_2max_; HR and RPE (self-report) Physical activity: self-report Other: walking economy
Samuels et al. [6], USA	Total: 50/29 Accumulated: 15/12 Continuous: 17/17	Description: inactive adults. Sex: 81.4% F. Mean (SD) age: 50.3 ± 9.6 years. Mean (SD) BMI: 28.6 ± 5.1 kg/m^2^	Intervention duration: 4 weeks Mode: walking Intensity: moderate Setting: home-based Format: individual, unsupervised Progressive: no Accumulated condition: 7 days × ≤ 3 × ≥ 10 min Continuous condition: 7 days × 1 × 30 min Control condition: no control	Days per week walking goal was met: Accumulated: 2.3 days/week Continuous: 2.8 days/week	Physical activity: pedometer, accelerometer Other: Physical Activity Self-Efficacy Scale
Schachter et al. [38], Canada	Total: 143/143 Accumulated: 56/56 Continuous: 51/51 Control: 36/36	Description: sedentary women with fibromyalgia. Sex: 100% F. Mean (SD) age: 41.9 ± 7.7 years	Intervention duration: 16 weeks Mode: low-impact aerobics to music Intensity: final intensity 65–75% HRR Setting: home-based Format: individual, unsupervised Progressive: yes, target exercise duration met by week 11 Accumulated condition: 3–5 days × 2 × 15 min Continuous condition: 3–5 days × 1 × 30 min Control condition: maintain sedentary lifestyle, monthly meetings to discuss experiences of fibromyalgia. No educational content. Telephone contact every 4 weeks. (attention wait-list control)	% of prescribed exercise completed in each phase: Accumulated: (1) 46%, (2) 40%, (3) 42%, (4) 22% Continuous: (1) 68%, (2) 74%, (3) 54%, (4) 41%	CR fitness: *V*O_2max_ Physical activity: log book Other: pain, Chronic Pain Self-Efficacy Scale, Fibromyalgia Impact Questionnaire, Arthritis Impact Measurement Scale, Physician Rating of Disease Severity
Schmidt et al. [31], USA	Total: 48/38 Accumulated 1: 12/10 Accumulated 2: 12/8 Continuous: 12/12 Control: 12/8	Description: non-exercising, overweight female college students. Sex: 100% F. Mean (SD) age: 19.7 ± 1.4 years. Mean (SD) BMI: 31.4 ± 3.4 kg/m^2^	Intervention duration: 12 weeks Mode: cycle ergometer Intensity: 75% HRR + 5 bpm Setting: research facility Format: individual, unsupervised Progressive: yes, target exercise duration met by week 5 Accumulated conditions : 5 days × 3 × 10 min or 5 days x 2 x 15 mins Continuous condition: 5 days × 1 × 30 min Control condition: asked to maintain their normal activity routine throughout the study Self-monitored, calorie-restricted diet (total calorie intake 80% of REE)	Number of sessions attended per week: Accumulated: attended sessions 3.7 days/week (average) Continuous: attended sessions 3.9 days/week	Anthropometrics: mass, BMI, SKF, HC, WC, thigh and upper arm circumference CR fitness: *V*O_2max_ Physical activity: pedometer Other: total calories
Serwe et al. [39], USA	Total: 60/53 Accumulated: 20/17 Continuous: 20/17 Control: 20/19	Description: inactive premenopausal female healthcare workers. Sex: 100% F. Mean (SD) age: 37.2 ± 7.5 years. Mean (SD) BMI: 29.1 ± 8.6 kg/m^2^	Intervention duration: 8 weeks Mode: walking Intensity: 60–70% HRR Setting: home-based Format: individual, unsupervised Progressive: none Accumulated condition: 5 days × 3 × 10 min Continuous condition: 5 days × 1 × 30 min Control condition: asked to maintain normal physical activity levels and diet during intervention period	% of prescribed sessions: Accumulated: 69% Continuous: 80%	Anthropometrics: mass, BMI, WC, HC CV outcomes: resting SBP, resting DBP CR fitness: resting HR, 6-min walk test Physical activity: pedometer, log book
Shiau et al. [43], Taiwan	Total: 20/20 Accumulated: 10/10 Continuous: 10/10	Description: young male adults (juniors in a military college). Sex: 0% F. Mean (SD) age: 20.0 ± 1.0 years. Mean BMI: 24.3 kg/m^2^. Mean BF%: 17.1%	Intervention duration: 10 weeks Mode: resistance training Intensity: 50–75% of 1 RM for 15 to 8 reps Setting: military college Format: group, supervision not reported Progressive: yes Accumulated condition: 3 days × 3 × 10 min (1 set of each exercise per session) Continuous condition: 3 days × 1 × ~ 30 min (3 sets of each exercise per session) Control condition: no control	NR	Anthropometrics: BMI, FM, BF%, FFM via BIA Strength: 1 RM bench press; 30-s Wingate test: peak power output during any 5-s period; average power output for total 30 s; and fatigue index; blood lactate concentrations at 3rd, 5th, 15th, and 30th min post-Wingate test

*APHR* age-predicted heart rate, *BF%* body fat percentage, *BIA* bioelectrical impedance analysis, *BMI* body mass index, *CR* cardiorespiratory, *CV* cardiovascular, *DBP* diastolic blood pressure, *F* female, *FFM* Fat free mass, *FM* Fat mass, *FPG* fasting plasma glucose, *FPI* fasting plasma insulin, *HC* hip circumference, *HDL-C* high-density lipoprotein cholesterol, *HOMA-IR* Homeostasis Model Assessment of Insulin Resistance, *HR* heart rate, *HR*_*max*_ heart rate maximum, *HRR* heart rate reserve, *LDL-C* low-density lipoprotein cholesterol, *MET* metabolic equivalent, *NR* not reported, *REE* resting energy expenditure, *RM* repetition maximum*, RPE* rate of perceived exertion, *SD* standard deviation*, SBP* systolic blood pressure, *SKF* sum of skinfold, *TBARS* thiobarbituric acid reactive substances, *VO*_*2max*_ maximal oxygen uptake, *WC* waist circumference, *WHR* waist-to-hip ratio

### Study Design

After screening, we were left with 19 eligible trials. Two trials [25, 26] had associated publications—three papers each [25–30]. Fifteen trials adopted a randomised parallel design, whereas, two trials were non-randomised parallel trials [31, 32] and the other two trials adopted a randomised cross-over design [33, 34]. Of the 17 parallel design trials, all allocated participants to either an accumulated or continuous exercise group, but only nine included a control arm [26, 29, 31, 35–40]. None of the trials with cross-over included a control condition.

Three trials involved two accumulated exercise interventions [31, 41, 42]. One of these trials investigated the effects of accumulating 30 min of exercise on 6 days/week by performing either three 10-min bouts/day or in any combination of bouts the participants chose as long as each bout was at least 5 min [41]. In another trial, participants in two accumulated exercise groups were given the same exercise prescription, but in one group participants were given a treadmill to perform the exercise in their homes [42], whereas in the remaining trial, Schmidt et al. compared the effects of three 10-min bouts of cycling exercise with two 15-min bouts performed on 5 days/week [31].

The 19 eligible trials involved 1080 randomised participants; 480 participants were allocated to an eligible accumulated exercise intervention, 398 participants to an eligible continuous exercise, and 178 to an eligible control. The median (minimum–maximum) sample size was 47 (18–148) participants for trials, 17 (8–56) for accumulated exercise groups, 16 (10–51) for continuous exercise, and 15 (10–45) for control. Only three trials had group sample sizes above 30 participants [25, 31, 42].

### Participant Characteristics

Except for the Schachter et al. [38] study, which evaluated participants with fibromyalgia, all trials included participants who were disease-free, and excluded those with a history of cardiovascular or metabolic disease, medical problems, or those who were taking medication known to affect health factors such as heart rate, blood pressure, or lipid profile. The average (SD) age of participants across the 19 trials was 40 (9.6) years. All eligible trial samples comprised adults, although, two trials involved young adults [31, 43]. Nine of the eligible trials comprised only female participants: four involved women who were premenopausal or under 50 years of age [31, 39, 42, 44], two consisted of postmenopausal women who were hormone replacement therapy users or non-users [25], one involved middle-aged women who were obese [40], one involved women aged between 31 and 57 years [35], and one trial was composed of women with fibromyalgia aged 20–55 years [38]. Three trials had male only samples [43, 45, 46], whereas the remaining seven had samples including both male and female participants [6, 32–34, 36, 37, 41]. Most of the participants in these trials with both genders were female (mean ± SD percentage of female = 67 ± 15%).

Only four trials provided ethnicity data, and all reported a large majority of white participants (mean = 95% [6, 37, 38, 41]). Nine of the trials were conducted in the US [6, 31, 32, 34, 39, 41, 42, 44, 45], three were Northern Irish [33, 35, 36], two were Canadian-based [37, 38], while one each were carried out in Iran [26], Japan [46], Finland [25], South Korea [40], and Taiwan [43].

Nearly all trials described participants as either sedentary [25, 26, 33, 37, 38, 41, 42, 44–46], inactive [6, 36, 39], non-exercising [31], not regularly exercising [40], or untrained [32]. However, because trials used different definitions for the terms sedentary and inactive, there is likely to be some variation in the baseline physical activity of participants. One study described participants as low to moderately active [34], whereas another trial reported participants as having no resistance training experience but engaging in ball sports 3–4 times per week [43]. Twelve trials provided baseline cardiorespiratory fitness data, of which ten reported relative *V*O_2max_ (mean ± SD 30.6 ± 4.7 ml/kg/min) and two trials included absolute *V*O_2max_ values [31, 44].

In five of the trials, participants were described as overweight or obese [26, 31, 40, 42, 44]. Based on baseline data from 18 trials (only Schachter et al. [38] did not provide data), the mean (SD) mass of participants was 76 (9) kg. The average (SD) BMI of participants in the eligible trials was 28.0 (3.2) kg/m^2^, which is categorised as overweight (*n* = 12 trials [6, 25, 26, 31–33, 35, 39, 41, 42, 44, 46]).

### Intervention and Control Group Characteristics

The median duration of interventions was 12 weeks. The shortest duration intervention was 4 weeks [32], and only one trial consisted of an intervention lasting over 20 weeks [42] (72 weeks). Across 16 accumulated and continuous exercise groups, the median total prescribed dose of exercise was 1320 min or 110 min per week. We were unable to calculate the exact dose of exercise given to participants in four trials. The potential minimum and maximum ranges of exercise dose in two of these trials were 13,600–14,400 min [42] and 1176–1694 min [38]. In another trial [25], exercise dose was described as total energy expended during exercise rather than minutes of exercise; the exercise dose in this study was 22,500 kcal in total or 1500 kcal/week. Finally, the median (minimum–maximum) number of sessions prescribed for the accumulated and continuous exercise interventions was 120 (60–1440) and 48 (20–360), respectively.

#### Intervention Mode

Most of the trials employed walking interventions (*n* = 12, [6, 25, 26, 33, 35–37, 39–42, 44]). Two trials each chose indoor cycling [31, 46] and jogging [32, 45] as their exercise mode, whereas one trial each used low-impact aerobics to music [38] and a variety of aerobic exercise modes including walking, jogging, cycling, cross-country skiing, rowing, and stair-climbing machines [34]. Only one trial [43] used resistance training (via resistance equipment) as the mode of exercise.

#### Intervention Intensity

All exercise intensities set in the eligible trials would be considered moderate. Twelve of the trials set intensity relative to a percentage of the participant’s maximal heart rate (MHR) or heart rate reserve (HRR) (i.e. MHR minus resting heart rate). Of these trials, seven [25, 31, 32, 34, 35, 37, 45] used a directly measured MHR in their calculation of a relative target heart rate, whereas the remaining five [26, 33, 38, 39, 42] used age-predicted MHR (i.e. 220 minus age). The percentage heart rate employed by the 12 trials ranged between 60 and 80% of MHR or HRR. Two trials each prescribed exercise intensity based on the rate of perceived exertion (RPE)—one used a modified 0–10 Borg scale [41] and the other used the 6–20 Borg scale [38]. Two trials prescribed intensity based on a percentage of *V*O_2max_. Eguchi et al. [46] instructed participants to cycle at a power output (W) corresponding to 50% of their directly measured *V*O_2max_, whereas Chung et al. [40] asked participants to walk on a treadmill at an intensity corresponding to 83% of estimated *V*O_2max_ so that participants expended 200 kcal in 30 min of walking. One trial [43] set exercise intensity according to the number of repetitions of a resistance exercise performed at a certain percentage of 1 repetition maximum (1 RM). Two trials did not mention specific exercise intensity but prescribed ‘brisk’ walking [36, 42], whereas another trial reported only that the walking was ‘moderate’ intensity.

Only four trials used intensity to apply progression to their intervention [34, 38, 43, 46], and of these, only two provided specific details (Schachter et al. [38], from 40–50% to 65–75% HRR and 10–11 to 13–14 RPE; Quinn et al. [34], from 50–60% to 70–80% HRR). Eight trials, however, applied progression by increasing exercise time per week through increasing days/week, minutes/bout, or bouts/day over the intervention period [25, 26, 31, 37, 38, 41, 42, 44]. Shiau et al. [43] increased the load (% 1 RM) participants lifted in each exercise session to provide progression. Only one trial [25] did not provide details about the specific progression applied.

#### Accumulated Exercise Bout Number and Duration, and Exercise Frequency

In the 22 accumulated exercise interventions included in the 19 eligible trials, the most common bout duration was 10 min (*n* = 15). Of these 15 interventions, one prescribed two bouts/day [36], 11 set three bouts/day [31–33, 35, 37, 39–41, 43, 45, 46], and three prescribed four bouts/day [42, 44]. In all but four of these interventions, frequency was set at 5 days/week (Chung et al. [40], 3 days/week; Eguchi et al. [46],  3 days/week; Shiau et al. [43], 3 days/week; Murtagh et al. [36], 3 days/week). Three trials consisted of two bouts of 15 min/day performed on 3–5 [38], 4 [34], and 5 [31] days/week. In the remaining interventions, one asked participants to accumulate 40 min/day of exercise in three bouts/day on 5 days/week [26]; another intervention directed participants to accrue 30 min of exercise in at least 10-min bouts (two to three bouts/day) daily [6]; similarly, in one of Coleman et al.’s [41] intervention groups, participants were told to perform bouts of at least 5 min to amass 30 min of exercise on 6 days/week; and finally another intervention had participants complete two bouts of exercise of a sufficient duration to expend 150 kcal per bout 5 days/week [25]. Shiau et al. [43] asked participants to perform one set each of nine resistance exercises, with 30-s recovery between sets, in three sessions (~ 10 min per session performed at 8 am, 5 pm and 9 pm) on 3 days/week.

#### Continuous Exercise Bout Duration and Exercise Frequency

The most common continuous exercise prescription was 30 min of exercise on 5 days/week (150 min/week total; *n* = 7 [31–33, 35, 37, 39, 45]), followed by 40 min on 5 days/week (200 min/week total; *n* = 3 [26, 42, 44]). The largest amount of weekly exercise prescribed to participants was 7 days of 30 min (210 min/week total, [6]), whereas the least prescribed was 3 days of 20 min/week (60 min total [36]). One trial [25] did not prescribe exercise based on time; instead they gave participants an energy expenditure target of 300 kcal (1256 kJ) per exercise session. In another trial [43], participants performed three sets each of nine resistance exercises (30-s and 60-s rest between sets and exercises, respectively) at 5 pm on 3 days/week.

#### Intervention Format and Setting

Only three trials consisted of completely supervised exercise sessions [31, 32, 40], whereas three trials included a mixture of supervised and unsupervised sessions [25, 35, 36]. It was unclear in one trial [43] whether participants were supervised. The exercise sessions were unsupervised in all other trials. Two of the supervised interventions took place in a university exercise facility, whereas the other one [40] was performed in a public health centre. The mixed supervision interventions occurred in a university gym [36] or campus [35], or in both an indoor track and outdoors [25]. For the unsupervised interventions, most were home-based (i.e. participant’s own house—including its surrounding areas, such as nearby streets, parks, etc.). Participants in one trial performed their exercise sessions in a military college fitness facility [43]. Eight trials consisted of home-based outdoor walking [6, 26, 33, 37, 39, 41, 42, 44]. One trial asked participants to exercise at home by following instructions on an aerobics video [38], whereas another trial gave treadmills to participants in one of the groups, so they could walk at home [42]. In a trial consisting of Japanese workers, each worker had access to a cycle ergometer placed within 5 min of their workplace [46]. DeBusk et al. [45] allowed participants to complete their prescribed jogging either at home or at work, whereas Quinn et al. [34] permitted participants to complete the exercise sessions using a variety of aerobic modalities in presumably a variety of different settings. All interventions were delivered in an individual format, except for two trials that involved a group exercise intervention [31, 43].

In addition to an exercise intervention, five trials included dietary modification, in the form of hypocaloric diets. The calorie-restricted diets in these trials consisted of 500 kcal less than participants’ Harris Benedict equation-derived daily energy intake [26], 300 kcal/day less than ‘usual intake’ [40], total calorie intake of 80% of resting energy expenditure [31], a target calorie intake of 1200–1500 kcal/day with fat limited to 20% of total intake [44], and 1500 kcal target intake for participants weighing at least 90 kg, and 1200 kcal for those weighing less than 90 kg [42]. The calorie restrictions were applied to all conditions in each trial. One additional trial [43] provided participants with nutrition instruction (recognition and recording of food categories and portions) from a nutritionist.

Six trials (35%) also provided an additional education or behaviour change component to their exercise arms. These components took the form of educational classes held at the beginning of the trials [26, 41], weekly or monthly meetings [38, 41, 42, 44], and phone calls during the study period [38, 39].

#### Control Group Characteristics

Of the nine (47%) trials that included a control group, seven trials asked control participants to maintain their usual activity routine throughout the study [26, 31, 33, 37–39] [25, 40]. One trial did not report what advice was given to controls [35]. Three of the control groups could be described as attention controls, given that study personnel contacted participants during the study period [25, 37, 38].

### Outcomes

Three trials reported *V*O_2max_ as the primary outcome [25, 37, 38], whereas one trial each included change in percentage body fat [40], 1 RM bench press [43], physical activity [39] and weight loss [42] as primary outcomes. Most trials, however, did not specify their primary outcome measure. Cardiorespiratory fitness was included as an outcome measure in the majority of eligible trials. Seven trials measured *V*O_2max_ using a maximal fitness test [25, 31, 34, 35, 37, 38, 45]. Sub-maximal testing was employed in seven included trials [33, 39, 41, 42, 44, 46]. One trial [43] reported muscular strength and anaerobic performance (via a 30-s Wingate test) as outcomes. Ten trials measured resting systolic (SBP) and diastolic blood pressure (DBP) of participants [25, 33, 35, 36, 39–41, 44–46], and resting heart rate was reported in two trials [44, 46].

Blood lipid measures (including total cholesterol, low-density lipoprotein (LDL) cholesterol, HDL cholesterol, and triglycerides) were measured in seven trials [25, 33, 34, 36, 40, 46]. Three trials also measured fasting blood glucose [25, 40, 46], and two of these trials also reported fasting insulin [25, 46].

Body mass was reported in all but five trials [6, 34, 37, 38, 43]. Body fat percentage was assessed in 13 trials, either by sum of skinfolds [25, 26, 31–35, 37], bioelectrical impedance analysis [26, 36, 40, 41, 43], or dual energy X-ray absorptiometry [42]. Fat-free mass was assessed in four trials [34, 40, 42, 43].

Most trials included a measure of exercise levels, but exercise was only reported as an outcome measure in ten trials. Self-reported methods, including questionnaires and diaries, were used alone in five trials [26, 33–35, 45]. In addition to using self-reported methods, four trials tracked exercise with pedometers [6, 25, 38, 39], and in two trials participants wore accelerometers [6, 41]. In another trial [31], the number of miles walked per week was monitored using pedometers to account for physical activity beyond the prescribed exercise programmes. Shiau et al. [43] recorded participants’ habitual physical activity outside of the prescribed exercise sessions via the International Physical Activity Questionnaire (IPAQ).

Dietary intake was measured in six trials [25, 26, 32, 42–44]. A few included trials also reported outcomes for a range of psychological parameters, including mood [33, 37] and self-efficacy [33, 34].

### Excluded Studies

Of the 37 trials we excluded after full-text screening, most (*n* = 19) were excluded as they did not compare a single bout of exercise with the same intensity and mode of exercise of the same total duration, accumulated in multiple bouts over the course of a day. Several of these trials attempted to hold volume constant between exercise groups, resulting in different intensities or duration of exercise in each group. Ten trials were ineligible due to study design. Of the remaining ineligible trials, four only had an abstract available, one was not a journal article, two were not published in English, and one included participants that were under 18 years of age.

### Risk of Bias

**Fig. 2:**
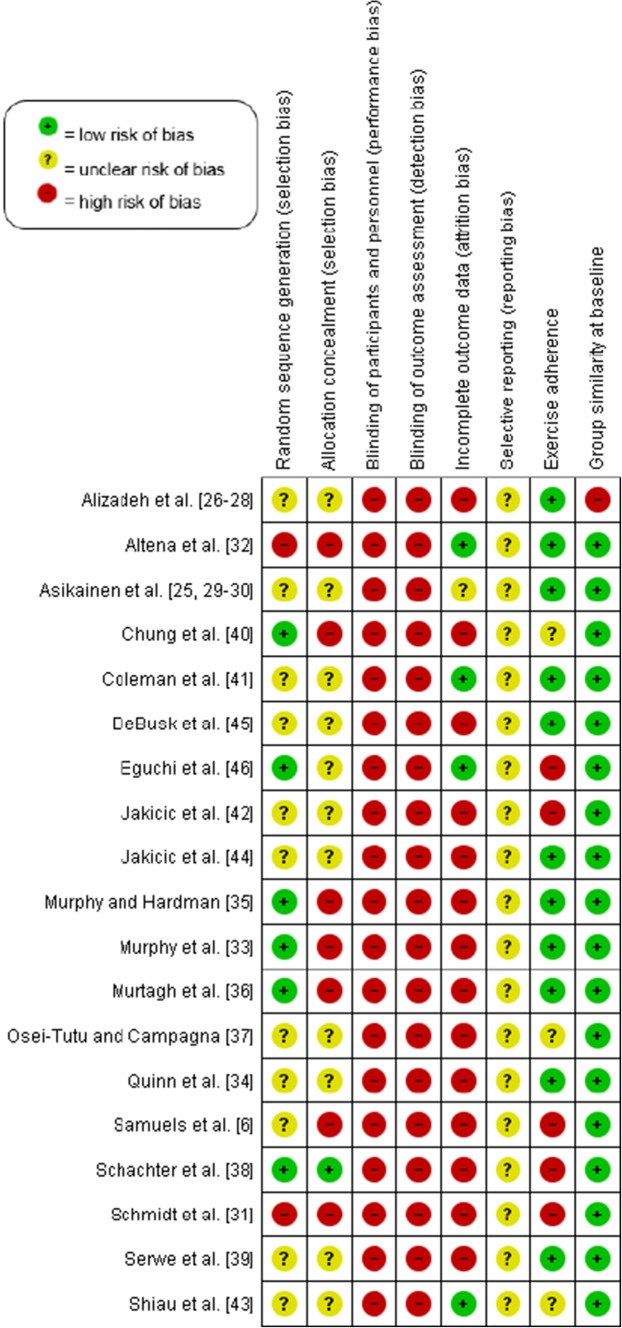
Risk of bias summary

**Fig. 3:**
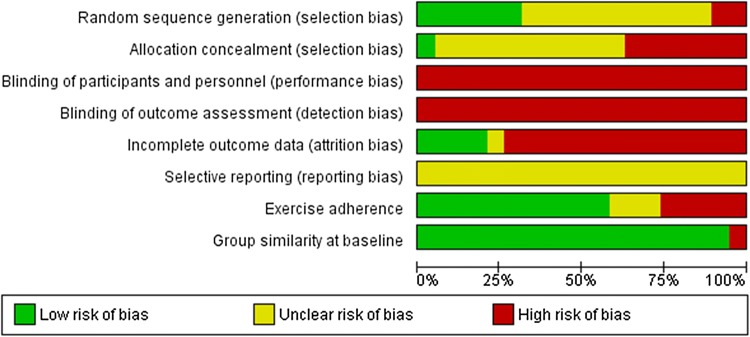
Risk of bias graph

The risk of bias judgement for each domain for individual trials is presented in Fig. 2, and an overall summary of the risk of bias can be found in Fig. 3 (see Electronic Supplementary Material Appendix S2 for judgement details). All trials were categorised as ‘trials at a high risk of bias’ and no trial was considered as a ‘trial at less than high risk of bias’ compared with other trials at high risk of bias. Therefore, no sensitivity analysis was possible for any of the outcomes.

#### Allocation

Only one trial [38] was considered as having a low risk of selection bias, because the authors adequately generated their randomised sequence with a random component and adequately concealed allocation to the intervention so that participants and investigators could not foresee assignment to the trial conditions. Another five trials (26%) used a random component to assign participants to study conditions, but four of these trials [33, 35, 36, 43] were at a high risk of selection bias, because participants or investigators might have foreseen assignment to the study groups; the other trial [46] was at an unclear risk of bias due to insufficient information being provided.

Two trials used a non-random component to generate the sequence [31, 32] and participants or investigators could foresee group allocation, and were thus judged to be at a high risk of selection bias. One further trial [6] was at a high risk of selection bias because one of the authors oversaw randomisation and would have foreseen group assignment. We judged ten trials (53%) to have an unclear risk of selection bias, because they lacked descriptions of both the generation of the random sequence and the allocation concealment method.

#### Blinding

All eligible trials included were at high risk for performance bias because it is not possible to blind the trial personnel and participants to exercise interventions. Similarly, all trials were considered at a high risk of detection bias because they either failed to blind outcome assessors or gave no information on blinding of all outcome assessors—which we judged as lack of blinding. However, two trials [33, 35] did employ outcome assessors blinded to participants’ group assignment to measure blood pressure. Outcome assessors were blinded to group allocation in only one of 19 trials [32] that assessed anthropometric measures.

#### Incomplete Outcome Data

Of the 1080 randomised participants who participated in the eligible trials, 17% dropped out before the end of the intervention period. Drop-outs were slightly higher in the accumulated exercise bouts conditions (20%) compared with continuous exercise (16%) and control (14%).

Only four trials (21%) were at a low risk of attrition bias; for three trials this was because they retained all participants to the end of the intervention period [32, 40, 46], whereas the other trial reported just one drop-out during the intervention [41]. One trial was considered at an unclear risk of attrition bias because although an intention-to-treat analysis was reported, the authors provided no information about how missing data were handled [25]. All other trials were considered at a high risk of attrition bias due to either high attrition (≥ 20% [6, 26, 33–38, 40, 42, 47]) or inappropriate handling of missing data [39, 42, 44, 45].

#### Selective Reporting

We judged all 19 trials as having an unclear risk for reporting bias, as no study protocol, design paper, or trial registration was available; the information was therefore insufficient to judge this item for eligible trials.

#### Baseline Imbalances

Eighteen trials (95%) were at low risk of bias owing to adequate group similarity at baseline, and one was at high risk of bias, because the baseline BMI differed across groups [26].

#### Exercise Adherence

In 12 trials (637 participants) that reported the percentage of total prescribed exercise sessions or time completed [25, 26, 33–36, 38, 39, 42, 44–46], the mean percentage adherence was lower in the accumulated exercise condition compared with continuous exercise (78 ± 17% vs. 83 ± 15%). Participants adhered adequately to the exercise intervention in 11 (61%) of the trials; however, in five trials (28%), adherence to the exercise intervention was so low that we judged it to cause a high risk of bias [6, 31, 38, 42, 46]. In one of these trials [6], participants met their daily goal of 30 min on only 2.3 days and 2.8 days per week for accumulated and continuous exercise, respectively, whereas another [31] reported that participants exercised on an average of 3.7 and 3.9 days of a target of 5 days a week for the respective conditions. Finally, three trials were judged to be at an unclear risk of bias because they provided no adherence data [37, 40, 43].

### Effects of Interventions: Accumulated Versus Continuous Exercise and Accumulated Exercise Versus Control

Full results of our meta-analysis can be found in Electronic Supplementary Material Appendix S3.

#### Exercise Adherence

Meta-analysis was possible for comparisons between accumulated and continuous exercise for total minutes of exercise, percentage prescribed sessions completed, average days per week of exercise, self-reported exercise (overall, minutes per week, and minutes per day), objectively measured exercise (overall and pedometer measured only), heart rate (overall and average heart rate in bpm), and RPE. Of these outcomes, we found that continuous exercise groups completed a statistically higher percentage of prescribed sessions (MD − 3.88%, 95% CI − 6.92 to − 0.84; *I*^2^ = 46%; eight studies, 384 participants), but the accumulated exercise group achieved statistically greater amounts of exercise when it was objectively measured (SMD 0.25, 95% CI 0.01–0.49, *I*^2^ = 25%; six studies, 523 participants).

Subgroup analyses revealed that the effect on the percentage of prescribed sessions completed in favour of continuous exercise was only statistically significant for trials employing an exercise dose of 150 min (MD − 3.07, 95% CI − 4.47 to − 1.68, *I*^2^ = 0%; three studies, 85 participants), and not those which prescribed fewer or greater than 150 min. However, the statistically higher objectively measured exercise in accumulated exercise groups was evident only in trials that prescribed an exercise dose of > 150 min (SMD 0.33, 95% CI 0.04–0.61, *I*^2^ = 31%; four studies, 420 participants). No accumulated exercise versus control meta-analysis was possible due to too few trials reporting exercise levels in the control groups.

#### Cardiorespiratory Fitness

We found no statistical differences between accumulated and continuous exercise for any cardiorespiratory fitness outcomes (*V*O_2max_, relative *V*O_2max_, exercise economy, and test duration/distance) in our analyses of change from baseline scores to post-intervention values.

However, there was a moderate favourable effect on post-intervention *V*O_2max_ values with accumulated exercise compared with control (SMD 0.52, 95% CI 0.24–0.81, *I*^2^ = 6%; four studies, 223 participants). Similarly, we found statistically higher relative *V*O_2max_ post-intervention values (MD 2.32 ml/kg/min, 95% CI 1.10–3.54, *I*^2^ = 4%; three studies, 197 participants) and statistical improvements in relative *V*O_2max_ from baseline to post-intervention (MD 2.78 ml/kg/min, 95% CI 2.51–3.05, *I*^2^ = 0%; two studies, 110 participants) with accumulated exercise versus control. However, there was no difference between accumulated exercise and controls when exercise economy and test duration or distance outcomes were pooled.

No statistical differences were observed in any subgroup analysis performed by exercise dose (< 150 min, 150 min, and > 150 min) in comparisons between accumulated exercise and either continuous exercise and control.

Additional cardiorespiratory fitness outcomes not included in the meta-analysis were as follows: Murtagh et al. [36] observed statistical pre- to post-intervention reductions in heart rate at stages 2 and 3 of a treadmill test (*p* < 0.05) and mean RPE (*p* < 0.05) in both accumulated and continuous exercise groups, but not in the control group. However, the authors reported no differences between groups for changes in mean *V*O_2_ during a submaximal treadmill test pre- to post-intervention. Another study [34] found that after 12 weeks of training, walking economy (% *V*O_2max_ and % heart rate maximum while walking at 101.8 m/min) improved statistically (*p* < 0.05) in an accumulated exercise group, but not in a continuous exercise group. Statistical pre- to post-intervention reductions in SBP and DBP at 101.8 m/min walking speed were observed in the accumulated exercise group (*p* < 0.05), whereas only reductions in DBP were reported in the continuous exercise group (*p* < 0.05). Murphy and Hardman [35] found statistically increased *V*O_2_ at 2 mmol/l in both accumulated and continuous exercise groups relative to controls (both *p* < 0.05), but no between exercise group differences. Another study [45] found that accumulated and continuous exercise similarly statistically decreased peak (both *p* < 0.01) and submaximal exercise (both *p* < 0.001) heart rate, but found no statistical changes in blood pressure during submaximal or maximal exercise in either group. Finally, one study [44] reported no pre- to post-intervention improvements in predicted *V*O_2_ at heart rates of 110 and 125 bpm for both accumulated and continuous exercise groups (both *p* < 0.001), but no statistical between-group differences were found.

#### Resting Cardiovascular Outcomes

In meta-analyses of resting heart rate (post-intervention) and SBP and DBP (post-intervention and change from baseline) values, we found no statistical differences between accumulated and continuous exercise.

Compared with control, accumulated exercise was associated with statistically lower post-intervention DBP values (MD − 4.83 mmHg, 95% CI − 7.83 to − 1.84, *I*^2^ = 26%; four studies, 161 participants), but no statistically different effects on SBP (post-intervention or change scores).

In comparisons between accumulated and continuous exercise and accumulated exercise and control, we found no statistical differences for any outcome by exercise dose.

#### Anthropometric and Body Composition Outcomes

We found a small but statistical reduction in body mass from baseline to post-intervention in favour of accumulated exercise compared with continuous exercise (MD − 0.92 kg, 95% CI − 1.59 to −0.25, *I*^2^ = 0%; five studies, 211 participants). Participants in the accumulated exercise groups, however, did not have statistically lower body mass post-intervention than the continuous exercise groups. No differences between accumulated and continuous exercise were found for any other anthropometric or body composition outcome.

Compared with control, accumulated exercise statistically reduced baseline to post-intervention values for body mass (MD − 1.94 kg, 95% CI − 3.42 to − 0.47, *I*^2^ = 82%; four studies, 97 participants), BMI (− 0.97 kg/m^2^, 95% CI − 1.70 to − 0.24, *I*^2^ = 79%; two studies, 48 participants), waist circumference (− 2.62 cm, 95% CI − 4.67 to − 0.56, *I*^2^ = 67%; two studies, 44 participants), and sum of skinfolds (− 6.39 mm, 95% CI − 8.25 to − 4.53, *I*^2^ = 0%; three studies, 70 participants). The removal of the most extreme value did not reduce heterogeneity in the body mass analysis above. A combined analysis of post-intervention and change scores revealed a statistical but small reduction in body fat percentage with accumulated versus continuous exercise (− 0.92%, 95% CI − 1.78 to − 0.07, *I*^2^ = 0%; four studies, 147 participants). No statistical differences between accumulated exercise and control were observed for post-intervention body mass, BMI, and waist or hip circumference values.

In subgroup analyses by exercise dose, accumulating > 150 min of weekly exercise in multiple bouts per day resulted in statistical small effects on body fat percentage (combined post-intervention and change from baseline values; MD − 0.87%, 95% CI − 1.71 to − 0.04, *I*^2^ = 0%; three studies, 166 participants) compared with 150 min of exercise amassed via single continuous bouts per day. Similarly, compared with control, accumulating 150 min/week of exercise in multiple bouts per day resulted in statistically lower body mass (post-intervention; − 3.01 kg, 95% CI − 4.34 to − 1.68, *I*^2^ = 73%; two studies, 52 participants) and sum of skinfolds (post-intervention; − 6.46 mm, 95% CI − 8.38 to − 4.54, *I*^2^ = 0%; two studies, 48 participants).

Additional anthropometric and body composition outcomes not included in the meta-analysis were as follows: Two studies found no statistical differences in body mass post-accumulated or continuous exercise [34, 45]. Quinn et al. [34] also reported no statistical difference between exercise groups for body fat or lean mass measured via six-site skinfold measurement and waist and hip circumferences. Another study [37] reported statistical pre- to post-intervention decreases (− 6.7%, *p* < 0.05) in body fat percentage in the continuous exercise group, but not in the accumulated exercise or control groups. Schmidt et al. [31] observed statistical within-group reductions in sum of circumferences (hip, waist, thigh, and upper arm) measures (*p* < 0.01) in accumulated and continuous exercise groups; however, no between-group differences were found. Finally, Jakicic et al. [42] reported no differences for bone mineral content between exercise groups.

#### Blood Biomarkers

Only seven trials (three with control groups [29, 36, 40]) reported blood biomarker data [29, 32–34, 36, 40, 46]. We found small statistical baseline to post-intervention reductions in LDL cholesterol with accumulated versus continuous exercise (MD − 0.39 mmol/l, 95% CI − 0.73 to − 0.06, *I*^2^ = 23%; two studies, 41 participants). No differences were observed for any other blood biomarker (total cholesterol, HDL cholesterol, triglycerides, fasting blood glucose, and fasting insulin). Compared with control, we found no statistical effects of accumulated exercise on any blood biomarker (total cholesterol, LDL cholesterol, HDL cholesterol, triglycerides, plasma glucose). No subgroup analyses by exercise dose were possible for any blood biomarker because too few trials were available.

Additional blood biomarker outcomes not included in the meta-analysis were as follows: One study [34] showed modest statistical improvements in HDL cholesterol (*p* < 0.05) with accumulated exercise only, but no changes in any other lipid values following either accumulated or continuous exercise. Another study [32] found no between-group differences in changes in any lipid outcome. Chung et al. [40] observed a statistical interaction effect between time and group (*p* < 0.01) for the atherogenic index [(total cholesterol − HDL cholesterol)/HDL cholesterol], with contrast analysis revealing statistical increases in the control group, but not in the two exercise conditions. Eguchi et al. [46] found no differences in oxidative stress (plasma thiobarbituric acid reactive substances) between accumulated and continuous exercise groups. Finally, another study [29] found statistically lower 2-h glucose concentrations (*p* < 0.05) in both accumulated and continuous exercise groups compared with control, but no differences between exercise groups. In the same study, no statistical differences in 2-h insulin were observed between exercise groups or control.

#### Psychological Outcomes

In meta-analyses involving only 41 participants in two studies [33, 37] that measured mood [both via profile of mood states (POMS)], continuous exercise resulted in statistically lower depression and anxiety subscale scores (SMD 0.93, 95% CI 0.15, 1.71, *I*^2^ = 27% and 0.68, 95% CI 0.03–1.32, *I*^2^ = 0%). However, no statistical differences in vigour subscale scores were found. Only one of these studies included a control group, so accumulated exercise versus control comparisons were not possible. Similarly, both studies prescribed an exercise dose of 150 min (both 5 days of 3 × 10-min bouts), so no subgroup analysis by exercise dose was possible. Only Osei-Tutu and Campagna [37] included the POMS total mood disturbance score and observed statistical decreases in both accumulated and continuous exercise groups over the 8-week intervention, but not in a control group. Conversely, only Murphy et al. [33] reported anger, confusion, and fatigue POMS subscale scores, and found no statistical differences in the effects of accumulated versus continuous exercise.

Three trials [6, 33, 38] compared the effects of accumulated and continuous exercise on self-efficacy-related outcomes. We did not combine these in a meta-analysis because of differences between the constructs assessed. Schachter et al. [38] found statistical improvements in self-efficacy for managing pain, managing other symptoms, and performing functional tasks among women with fibromyalgia who exercised in continuous bouts compared with controls (*p* = 0.034) at mid-intervention, and greater improvements in self-efficacy among accumulated bout exercisers compared with control (*p* = 0.001) at the end of the 12-week intervention. However, the effect on self-efficacy was similar between the two exercise patterns. Murphy et al. [33] found no changes in self-efficacy for walking among participants walking in accumulated or continuous exercise bouts, and increases in self-efficacy for cycling, jogging, and stair climbing among accumulated bout walkers (*p* < 0.05) only. The third trial [6] reported similar decreases in self-efficacy related to achieving physical activity recommendations among those assigned to accumulated and continuous bouts (*d* = 0.40 in both cases).

#### Other Outcomes not Included in the Meta-Analysis

Two trials [25, 38] compared the effects of accumulated and continuous exercise on pain-related outcomes using three self-report inventories (Fibromyalgia Impact Questionnaire, Body Pain Diagram, Arthritis Impact Measurement Scales 2) and physician-rated pain scores. In one trial [38], those assigned to the control group demonstrated improvements in pain (*p* = 0.046), whereas no differences were found within or between accumulated and continuous exercise bout groups. In the second trial, Asikainen et al. [25] measured self-reported exercise-related pain and injuries at the end of a 15-week intervention. Although 35% of exercise participants reported exercise-related pain, only 17% reported that the pain was sufficient to temporarily interrupt their participation. The authors [25] also observed that participants in the accumulated exercise bouts group reported statistically fewer lower-limb problems compared with continuous exercise group participants (*p* = 0.021).

No statistical differences were found for daily energy intake (kcal/day) or percentage of daily energy intake from fat between accumulated and continuous exercise, accumulated exercise and control, or any subgroup analysis by exercise dose. In one study [42] that did not report data, there were no statistical differences in pre- to post-intervention energy intake or macronutrient composition. Another study, by Alizadeh et al. [26], also reported no differences in changes in the percentage of energy from carbohydrate, fat, and protein post-accumulated versus continuous exercise.

Asikainen et al. [30] reported that the proportion of participants reaching maximum points on the one-leg squat test for lower-extremity muscle strength increased statistically in both accumulated and continuous exercise groups versus control (odds ratio 4.6 and 4.1 in accumulated and continuous groups, respectively, vs. control, *p* = 0.008). In the same trial, however, no between-group differences were observed in the proportion of participants reaching the maximum score on the one-leg standing balance test. Finally, walking time on the UKK 2-km Walk Test increased statistically in both exercise groups when compared with the control group (*p *< 0.001). Similarly, Shiau et al. [43] reported statistical improvements (*p* < 0.05) in maximal strength (via 1 RM bench press), anaerobic performance (via 30-s Wingate test), and blood lactate response to anaerobic exercise (30-s Wingate) after a 12 week accumulated (three bouts of one set of each exercise per session for 3 days/week) or continuous (three sets of each resistance exercise per session for 3 days/week) resistance training intervention, but no statistical between-group differences. DeBusk et al. [45] reported no statistical differences between accumulated and continuous groups for participant-reported sweating during exercise bouts, and overall enjoyment and convenience of exercise bouts. Finally, Shiau et al. [43] reported no statistical between-group differences in daily physical activity assessed via IPAQ.

## Discussion

This is the first meta-analysis considering the effects of splitting a continuous bout of exercise into shorter bouts of the same intensity and overall duration dispersed throughout the day. The majority of the studies included (16 of 19) were small (< 30 participants), and therefore, probably did not have sufficient power to detect changes in some outcomes. Pooling the weighted data in this analysis increases the power to detect such changes. The findings suggest that accumulating exercise in short bouts (at least 10 min) over the course of the day produces similar effects on a range of health-related outcomes, including cardiorespiratory fitness, blood pressure, lipids, and glucose metabolism, to performing the same exercise in one continuous bout. This strengthens the evidence base for current physical activity guidelines which suggest that short bouts are equivalent to longer continuous bouts.

Within our analysis, there is evidence from a small number of studies that accumulated bouts of exercise produce slightly more favourable changes in body mass and LDL cholesterol than continuous bouts of the same intensity and total duration. The mechanisms underlying potential differences in these effects on body mass have not been well elucidated. It is plausible, however, that the acute increase in metabolic rate induced by exercise [48] results in greater energy expenditure in two to three bouts compared to one bout, which over time amounts to a larger energy deficit and greater reductions in body mass. The larger decrease in LDL cholesterol following accumulated exercise compared to control can be linked to the alterations in body mass given the significant correlation between exercise induced decreases in body mass and reductions in fasting LDL cholesterol [49].

Accumulated exercise has often been promoted as a more palatable way of meeting physical activity recommendations. This suggestion is intuitively appealing given that time is often cited as a barrier to achieving sufficient daily physical activity [50]. However, the findings from this review do not support this assertion as there were higher levels of drop out from accumulated (20%) compared to continuous (16%) interventions. Although reasons given for drop-out were not extracted in this analysis, it seems likely that the additional constraint imposed on participants by requiring them to split an exercise bout into shorter bouts and to disperse them at specified intervals over the course of a day may have been more challenging than performing the required exercise in a real-life setting as a continuous intervention in an opportunistic and less regimented manner.

Walking was the mode of activity employed in most (63%, *n* = 12/19) of the studies comparing the effects of continuous and accumulated exercise. This may reflect both the accessibility of walking in terms of cost, skill requirement and acceptability among participants but it is also likely to be because unlike many other forms of exercise, walking can be easily incorporated into daily life. It is unlikely that someone choosing swimming or another facility-based exercise or one which requires a change of clothing would choose to split a continuous daily bout into multiple shorter bouts. This underscores the utility of walking as a flexible mode of exercise eminently suitable for helping people meet current physical activity guidelines. Of note is the predominance of female participants in the trials included in this study. This contrasts with the trends towards male participation in other exercise trials [51] and could reflect the greater acceptability of walking compared to other forms of activity among women as evidenced by the increased levels of self-reported recreational walking among women [52].

The trials included in this meta-analysis focused on cardiovascular and metabolic outcomes. Far fewer studies have compared the effects of continuous and accumulated bouts of exercise on psychological outcomes, such as self-efficacy and mood, which are likely to be important determinants of long-term adherence. Further comparisons of the effect of continuous and accumulated exercise on psychological outcomes are merited.

Although this review is a comprehensive summary of randomised controlled trials which have compared continuous and accumulated exercise, it is not without limitations. For the meta-analysis we pooled data from interventions which split continuous exercise into two, three, or four bouts per day and separated these bouts by typically 2–5 h, and so our conclusions cannot extend to a recommendation for the optimum way to spilt and disperse a continuous bout of exercise. Importantly, only one study included an intervention that consisted of bouts of less than 10 min, and we therefore cannot comment on the efficacy of accumulating exercise bouts of below that duration.

All of the studies included in this analysis were at a high risk of bias, and the low number of studies and relatively small sample sizes means that our estimates cannot be considered precise (using the Grades of Recommendation, Assessment, Development, and Evaluation (GRADE) definition) [53]. Many of the trials were reported poorly, with many failing to provide data on attrition, blinding of outcome assessors, and randomisation and allocation procedures. Finally, we restricted our search to English language publications, and may thereby have missed relevant trials comparing accumulated and continuous interventions.

## Conclusion

Our analysis suggests that splitting a continuous bout of exercise into shorter bouts of equivalent total duration dispersed over the course of a day does not alter its potential to evoke physiological effects likely to provide health benefit. Moreover, for weight loss, the fractionalisation of a single exercise bout into multiple bouts spread across the day may provide greater benefit. These findings provide further evidence that bout length, at least when bout duration is greater than 10 min, is not a determinant of the health effects associated with regular exercise.

## Electronic supplementary material

Below is the link to the electronic supplementary material.

Supplementary material 1 (DOCX 13 kb)

Supplementary material 2 (DOCX 18 kb)

Supplementary material 3 (DOCX 27 kb)

## Data Availability

Our full meta-analysis can be found at https://osf.io/7q9az/?view_only=970ed89a6a784a688834bc37f0929e06.
