# Supplementary material for: The Effects of Continuous Compared to Accumulated Exercise on Health: A Meta-Analytic Review
Source: Sports Med. 2019 Jul 2;49(10):1585–607. doi: 10.1007/s40279-019-01145-2 (PMC6745307; doi:10.1007/s40279-019-01145-2)
Supplement: Supplementary file 1 — Supplementary material 1 (DOCX 13 kb) [file 40279_2019_1145_MOESM1_ESM.docx]

**Electronic Supplementary Material Appendix S1: Search strategy**

**Pubmed search strategy (adapted for all other database searches):** ((((exercise[MeSH Terms]) OR (sports[MeSH Terms]) OR (resistance training[MeSH Terms]) OR (physical activity[MeSH Terms]) OR (walk*[Title/Abstract]) OR (exercis*[Title/Abstract]) OR (sport*[Title/Abstract]) OR (weight training[Title/Abstract]) OR (weightlifting[Title/Abstract]) OR (fitness[Title/Abstract]) OR (physical activit*[Title/Abstract]) OR (running[Title/Abstract]) OR (cycling[Title/Abstract]) OR (swimming[Title/Abstract])) AND ((single bout*[Title/Abstract]) OR (multiple bout*[Title/Abstract]) OR (short* AND bout*[Title/Abstract]) OR (long bout*[Title/Abstract]) OR (accumulat*[Title/Abstract]) OR (continuous[Title/Abstract]) OR (intermittent[Title/Abstract]) OR (repeated bout*[Title/Abstract]) OR (physical activity pattern*[Title/Abstract]) OR (exercise pattern*[Title/Abstract]))) AND (((randomized controlled trial[Publication Type]) OR (((((random*[Title/Abstract]) OR placebo*[Title/Abstract]) OR single blind*[Title/Abstract]) OR double blind*[Title/Abstract]) OR triple blind*[Title/Abstract]) OR ((retraction of publication[Publication Type]) OR retracted publication[Publication Type])) NOT (((((((((comment[Publication Type]) OR editorial[Publication Type]) OR meta-analysis[Publication Type]) OR practice-guideline[Publication Type]) OR review[Publication Type]) OR letter[Publication Type]) OR journal correspondence[Publication Type]) NOT randomized controlled trial[Publication Type]) OR ((((((random sampl*[Title/Abstract]) OR random digit*[Title/Abstract]) OR random effect*[Title/Abstract]) OR random survey[Title/Abstract]) OR random regression[Title/Abstract]) NOT randomized controlled trial[Publication Type])))) NOT ((animals[MeSH Terms]) NOT humans[MeSH Terms])
