# Supplementary material for: The Effects of Continuous Compared to Accumulated Exercise on Health: A Meta-Analytic Review
Source: Sports Med. 2019 Jul 2;49(10):1585–607. doi: 10.1007/s40279-019-01145-2 (PMC6745307; doi:10.1007/s40279-019-01145-2)
Supplement: Supplementary file 2 — Supplementary material 2 (DOCX 18 kb) [file 40279_2019_1145_MOESM2_ESM.docx]

**Electronic Supplementary Materials Appendix S2: Risk of bias judgements**

| Author | Randomisation (selection bias) | Allocation Concealment (selection bias) | Blinding of participants and personnel (performance bias) | Blinding of outcome assessment (detection bias) | Incomplete outcome data (attrition bias) | Selective reporting (reporting bias) | Exercise adherence | Group similarity at baseline |
| --- | --- | --- | --- | --- | --- | --- | --- | --- |
| Alizadeh et al. [26-28] | Low risk: "balanced block randomization in random blocks of 4, 6, and 8, into 2 groups" | Unclear: Method not given | High risk: Due to the nature of the intervention it was not possible to blind participants | High risk: Outcome assessors were probably not blinded to intervention allocation | High risk: High attrition and per protocol analysis | Unclear risk: trial was not preregistered and no published protocol was available. | Low risk: Adequate exercise adherence | High risk: On average, participants in the accumulated exercise group weighed less and had lower BMI's than the continuous and control groups. |
| Altena et al. [32] | High risk: Quasi-randomised, "alternatively assigned" | High risk: "alternatively assigned" | High risk: Due to the nature of the intervention it was not possible to blind participants | High risk: Outcome assessors were probably not blinded to intervention allocation | Low risk: No missing data | Unclear risk: trial was not preregistered and no published protocol was available. | Low risk: Adequate exercise adherence | Low risk: Groups were similar at baseline |
| Asikainen et al. [25, 29-30] | Unclear: Method no given "randomized separately in blocks of 15 subjects into the three groups" | Unclear: Method not given | High risk: Due to the nature of the intervention it was not possible to blind participants | High risk: Outcome assessors were probably not blinded to intervention allocation | Unclear: Intention-to-treat analysis claimed but details unclear | Unclear risk: trial was not preregistered and no published protocol was available. | Low risk: Adequate exercise adherence | Low risk: Groups were similar at baseline |
| Chung et al. [40] | Low risk: "block random sampling by the research coordinator" | High risk: "random sampling by the research coordinator" | High risk: Due to the nature of the intervention it was not possible to blind participants | High risk: Outcome assessors were probably not blinded to intervention allocation | High risk: Per protocol analysis | Unclear risk: trial was not preregistered and no published protocol was available. | Unclear risk: No adherence data reported | Low risk: Groups were similar at baseline |
| Coleman et al. [41] | Unclear: Method not given, ''randomly assigned' | Unclear: Method not given | High risk: Due to the nature of the intervention it was not possible to blind participants | High risk: Outcome assessors were probably not blinded to intervention allocation | High risk: Per protocol analysis | Unclear risk: trial was not preregistered and no published protocol was available. | Low risk: Adequate exercise adherence | Low risk: Groups were similar at baseline |
| DeBusk et al. [45] | Unclear: Method not given, ''randomly assigned' | Unclear: Method not given | High risk: Due to the nature of the intervention it was not possible to blind participants | High risk: Outcome assessors were probably not blinded to intervention allocation | High risk: Per protocol analysis | Unclear risk: trial was not preregistered and no published protocol was available. | Low risk: Adequate exercise adherence | Low risk: Groups were similar at baseline |
| Eguchi et al. [46] | Low risk: "randomly assigned using random numbers generated by a personal computer" | Unclear: Method not given | High risk: Due to the nature of the intervention it was not possible to blind participants | High risk: Outcome assessors were probably not blinded to intervention allocation | Low risk: No missing data | Unclear risk: trial was not preregistered and no published protocol was available. | Low risk: Low exercise adherence (accumulated vs. continuous: 56% vs. 69% of total sessions) | Low risk: Groups were similar at baseline |
| Jakicic et al. [44] | Unclear: Method not given, ''randomly assigned' | Unclear: Method not given | High risk: Due to the nature of the intervention it was not possible to blind participants | High risk: Outcome assessors were probably not blinded to intervention allocation | High risk: Per protocol analysis | Unclear risk: trial was not preregistered and no published protocol was available. | Low risk: Adequate exercise adherence | Low risk: Groups were similar at baseline |
| Jakicic et al. [42] | Unclear: Method not given, ''randomly assigned' | Unclear: Method not given | High risk: Due to the nature of the intervention it was not possible to blind participants | High risk: Outcome assessors were probably not blinded to intervention allocation | High risk: High attrition and baseline observation carried forward | Unclear risk: trial was not preregistered and no published protocol was available. | Low risk: Low exercise adherence (71% vs. 67% of total sessions) | Low risk: Groups were similar at baseline |
| Murphy & Hardman [35] | Low risk: Shuffled envelopes | High risk: member of research team randomised participants | High risk: Due to the nature of the intervention it was not possible to blind participants | High risk: Outcome assessors were probably not blinded to intervention allocation | High risk: High attrition and per protocol analysis | Unclear risk: trial was not preregistered and no published protocol was available. | Low risk: Adequate exercise adherence | Low risk: Groups were similar at baseline |
| Murphy et al. [33] | Low risk: Shuffled envelopes | High risk: member of research team randomised participants | High risk: Due to the nature of the intervention it was not possible to blind participants | High risk: Outcome assessors were probably not blinded to intervention allocation | High risk: High attrition and per protocol analysis | Unclear risk: trial was not preregistered and no published protocol was available. | Low risk: Adequate exercise adherence | Low risk: Groups were similar at baseline |
| Murtagh et al. [36] | Low risk: Shuffled envelopes "randomised on a two to one basis between training & control groups" | High risk: member of research team randomised participants | High risk: Due to the nature of the intervention it was not possible to blind participants | High risk: Outcome assessors were probably not blinded to intervention allocation | High risk: High attrition and per protocol analysis | Unclear risk: trial was not preregistered and no published protocol was available. | Low risk: Adequate exercise adherence | Low risk: Groups were similar at baseline |
| Osei-Tutu & Campagna [37] | Unclear: Method not given, "Participants were randomly assigned" | Unclear: Method not given | High risk: Due to the nature of the intervention it was not possible to blind participants | High risk: Outcome assessors were probably not blinded to intervention allocation | High risk: High attrition and per protocol analysis | Unclear risk: trial was not preregistered and no published protocol was available. | Unclear risk: No adherence data reported | Low risk: Groups were similar at baseline |
| Quinn et al. [34] | Unclear: Method not given, "randomized, crossover design" | Unclear: Method not given | High risk: Due to the nature of the intervention it was not possible to blind participants | High risk: Outcome assessors were probably not blinded to intervention allocation | High risk: Per protocol analysis | Unclear risk: trial was not preregistered and no published protocol was available. | Low risk: Adequate exercise adherence | Low risk: Groups were similar at baseline |
| Samuels et al. [6] | Unclear: Method not given, "used a randomized design with three conditions" | High risk: 2nd author initiated participant recruitment and oversaw randomization | High risk: Due to the nature of the intervention it was not possible to blind participants | High risk: Outcome assessors were probably not blinded to intervention allocation | High risk: Per protocol analysis | Unclear risk: trial was not preregistered and no published protocol was available. | Low risk: Low exercise adherence (2.3 vs. 2.8 d/wk completed of required "daily exercise") | Low risk: Groups were similar at baseline |
| Schachter et al. [38] | Low risk: "Random number sequence" | Low risk: randomisation was performed by researcher who was not connected with study | High risk: Due to the nature of the intervention it was not possible to blind participants | High risk: Outcome assessors were probably not blinded to intervention allocation | High risk: High attrition and baseline observation carried forward | Unclear risk: trial was not preregistered and no published protocol was available. | Low risk: Low exercise adherence (38 vs. 59% of total sessions) | Low risk: Groups were similar at baseline |
| Schmidt et al. [31] | High risk: "assigned (nonrandom)" | High risk: nonrandom | High risk: Due to the nature of the intervention it was not possible to blind participants | High risk: Outcome assessors were probably not blinded to intervention allocation | High risk: High attrition and per protocol analysis | Unclear risk: trial was not preregistered and no published protocol was available. | Low risk: Low exercise adherence (3.7 vs. 3.9 d/wk completed of required 5 d/wk) | Low risk: Groups were similar at baseline |
| Serwe et al. [39] | Unclear: Method not given, "8-week randomized controlled trial" | Unclear: Method not given | High risk: Due to the nature of the intervention it was not possible to blind participants | High risk: Outcome assessors were probably not blinded to intervention allocation | High risk: Baseline observation carried forward | Unclear risk: trial was not preregistered and no published protocol was available. | Low risk: Adequate exercise adherence | Low risk: Groups were similar at baseline |
| Shiau et al. [43] | Unclear: Method not given, ''randomly assigned' | Unclear: Method not given | High risk: Due to the nature of the intervention it was not possible to blind participants | High risk: Outcome assessors were probably not blinded to intervention allocation | Low risk: No missing data | Unclear risk: trial was not preregistered and no published protocol was available. | Unclear risk: No adherence data reported | Low risk: Groups were similar at baseline |
