# Supplementary material for: The Effects of Continuous Compared to Accumulated Exercise on Health: A Meta-Analytic Review
Source: Sports Med. 2019 Jul 2;49(10):1585–607. doi: 10.1007/s40279-019-01145-2 (PMC6745307; doi:10.1007/s40279-019-01145-2)
Supplement: Supplementary file 3 — Supplementary material 3 (DOCX 27 kb) [file 40279_2019_1145_MOESM3_ESM.docx]

**Electronic Supplementary Material Appendix S3: Meta-analysis results**

**S3.1 Accumulated vs. Continuous Exercise**

| **Outcome or Subgroup** | **Studies** | **Participants** | **Statistical Method** | **Effect Estimate** |
| --- | --- | --- | --- | --- |

| 1.1 Maximal Oxygen Uptake (post-intervention) | 9 | 439 | Std. Mean Difference (IV, Random, 95% CI) | -0.00 [-0.19, 0.19] |
| --- | --- | --- | --- | --- |
| 1.2 Relative Maximal Oxygen Uptake (post-intervention) [ml/kg/min] | 8 | 379 | Mean Difference (IV, Random, 95% CI [ml/kg/min]) | -0.50 [-1.53, 0.54] |

| 1.3 Maximal Oxygen Uptake (change) | 6 | 288 | Std. Mean Difference (IV, Random, 95% CI) | -0.07 [-0.56, 0.42] |
| --- | --- | --- | --- | --- |
| 1.4 Relative Maximal Oxygen Uptake (change) [ml/kg/min] | 4 | 125 | Mean Difference (IV, Random, 95% CI [ml/kg/min]) | 0.20 [-1.45, 1.86] |

| 1.5 Other Cardiorespiratory fitness | 6 | 285 | Std. Mean Difference (IV, Random, 95% CI) | -0.09 [-0.42, 0.25] |
| --- | --- | --- | --- | --- |
| 1.6 Exercise test duration | 4 | 228 | Mean Difference (IV, Random, 95% CI) | -15.36 [-67.73, 37.02] |

| 1.7 Resting Heart Rate (post-intervention) [bpm] | 2 | 92 | Mean Difference (IV, Random, 95% CI [bpm]) | -2.54 [-5.83, 0.76] |
| --- | --- | --- | --- | --- |
| 1.8 Resting Heart Rate (change) [bpm] | 1 | 23 | Mean Difference (IV, Random, 95% CI [bpm]) | -4.30 [-18.42, 9.82] |

| 1.9 Systolic Blood Pressure (post-intervention) [mmHg] | 7 | 274 | Mean Difference (IV, Random, 95% CI [mmHg]) | -1.28 [-4.00, 1.43] |
| --- | --- | --- | --- | --- |
| 1.10 Systolic Blood Pressure (change) [mmHg] | 3 | 99 | Mean Difference (IV, Random, 95% CI [mmHg]) | 0.56 [-5.19, 6.31] |

| 1.11 Diastolic Blood Pressure (post-intervention) [mmHg] | 7 | 274 | Mean Difference (IV, Random, 95% CI [mmHg]) | -1.27 [-3.09, 0.56] |
| --- | --- | --- | --- | --- |
| 1.12 Diastolic Blood Pressure (change) [mmHg] | 2 | 75 | Mean Difference (IV, Random, 95% CI [mmHg]) | 3.35 [-1.59, 8.29] |

| 1.13 Body mass (post-intervention) [Kg] | 8 | 314 | Mean Difference (IV, Random, 95% CI [Kg]) | 0.59 [-1.77, 2.94] |
| --- | --- | --- | --- | --- |
| 1.14 Body mass (change) [Kg] | 5 | 211 | Mean Difference (IV, Random, 95% CI [Kg]) | -0.92 [-1.59, -0.25] |

| 1.15 BMI (post-intervention) [kg/m2] | 7 | 351 | Mean Difference (IV, Random, 95% CI [kg/m2]) | 0.12 [-0.46, 0.70] |
| --- | --- | --- | --- | --- |
| 1.16 BMI (change) [kg/m2] | 3 | 72 | Mean Difference (IV, Random, 95% CI [kg/m2]) | -0.35 [-0.83, 0.13] |

| 1.17 Body Fat (post-intervention) | 8 | 342 | Std. Mean Difference (IV, Random, 95% CI) | 0.00 [-0.22, 0.22] |
| --- | --- | --- | --- | --- |
| 1.18 Body Fat % (combined) [%] | 8 | 340 | Mean Difference (IV, Random, 95% CI [%]) | -0.46 [-1.13, 0.21] |

| 1.19 Fat-free Mass (post-intervention) [Kg] | 3 | 192 | Mean Difference (IV, Random, 95% CI [Kg]) | 0.35 [-0.98, 1.68] |
| --- | --- | --- | --- | --- |
| 1.20 Waist Circumference (post-intervention) [cm] | 5 | 218 | Mean Difference (IV, Random, 95% CI [cm]) | 1.43 [-2.74, 5.60] |

| 1.21 Waist Circumference (change) [cm] | 3 | 66 | Mean Difference (IV, Random, 95% CI [cm]) | -0.38 [-1.65, 0.88] |
| --- | --- | --- | --- | --- |
| 1.22 Hip Circumference (post-intervention) [cm] | 3 | 72 | Mean Difference (IV, Random, 95% CI [cm]) | 2.32 [-2.65, 7.29] |

| 1.23 Waist-to-Hip Ratio (post-intervention) [cm] | 2 | 133 | Mean Difference (IV, Random, 95% CI [cm]) | 0.00 [-0.03, 0.03] |
| --- | --- | --- | --- | --- |
| 1.24 Sum of skinfolds (change) [mm] | 3 | 69 | Mean Difference (IV, Random, 95% CI [mm]) | -0.15 [-1.88, 1.57] |

| 1.25 Total Cholesterol (post-intervention) [mmol/L] | 4 | 157 | Mean Difference (IV, Random, 95% CI [mmol/L]) | 0.22 [-0.10, 0.54] |
| --- | --- | --- | --- | --- |
| 1.26 Total Cholesterol (change) [mmol/L] | 2 | 41 | Mean Difference (IV, Random, 95% CI [mmol/L]) | -0.09 [-0.75, 0.57] |

| 1.27 LDL-Cholesterol (post-intervention) [mmol/L] | 3 | 137 | Mean Difference (IV, Random, 95% CI [mmol/L]) | 0.50 [-0.36, 1.36] |
| --- | --- | --- | --- | --- |
| 1.28 LDL-Cholesterol (change) [mmol/L] | 2 | 41 | Mean Difference (IV, Random, 95% CI [mmol/L]) | -0.39 [-0.73, -0.06] |

| 1.29 Triglycerides (post-intervention) [mmol/L] | 4 | 158 | Mean Difference (IV, Random, 95% CI [mmol/L]) | 0.07 [-0.07, 0.20] |
| --- | --- | --- | --- | --- |
| 1.30 Triglycerides (change) [mmol/L] | 2 | 41 | Mean Difference (IV, Random, 95% CI [mmol/L]) | 0.00 [-0.06, 0.06] |

| 1.31 HDL-Cholesterol (post-intervention) [mmol/L] | 4 | 159 | Mean Difference (IV, Random, 95% CI [mmol/L]) | -0.06 [-0.23, 0.11] |
| --- | --- | --- | --- | --- |
| 1.32 HDL-Cholesterol (change) [mmol/L] | 2 | 41 | Mean Difference (IV, Random, 95% CI [mmol/L]) | 0.00 [-0.05, 0.05] |

| 1.33 Fasting Blood Glucose (combined) [mmol/L] | 3 | 136 | Mean Difference (IV, Random, 95% CI [mmol/L]) | -0.05 [-0.26, 0.16] |
| --- | --- | --- | --- | --- |
| 1.34 Fasting Insulin (combined) [mlU/L] | 2 | 112 | Mean Difference (IV, Random, 95% CI [mlU/L]) | 0.37 [-0.32, 1.06] |

| 1.35 Total Minutes of Physical Activity Completed [minutes] | 3 | 84 | Mean Difference (IV, Random, 95% CI [minutes]) | -20.31 [-93.97, 53.36] |
| --- | --- | --- | --- | --- |
| 1.36 Average days/week of Physical Activity [days/week] | 3 | 80 | Mean Difference (IV, Random, 95% CI [days/week]) | -0.26 [-0.59, 0.07] |

| 1.37 % Completed of Prescribed Sessions | 8 | 384 | Mean Difference (IV, Random, 95% CI) | -3.88 [-6.92, -0.84] |
| --- | --- | --- | --- | --- |
| 1.38 Self-reported Average Physical Activity | 7 | 363 | Std. Mean Difference (IV, Random, 95% CI) | 0.29 [-0.08, 0.66] |

| 1.39 Self-reported Physical Activity Min/week [min/week] | 5 | 244 | Mean Difference (IV, Random, 95% CI [min/week]) | 6.85 [-4.52, 18.22] |
| --- | --- | --- | --- | --- |
| 1.40 Self-reported Average Physical Activity [min/day] | 3 | 138 | Mean Difference (IV, Random, 95% CI [min/day]) | -0.51 [-4.77, 3.75] |

| 1.41 Objective Average Physical Activity | 6 | 523 | Std. Mean Difference (IV, Random, 95% CI) | 0.25 [0.01, 0.49] |
| --- | --- | --- | --- | --- |
| 1.42 Pedometer measured Physical Activity | 4 | 219 | Std. Mean Difference (IV, Random, 95% CI) | -0.00 [-0.44, 0.43] |

| 1.43 Heart Rate | 6 | 227 | Std. Mean Difference (IV, Random, 95% CI) | -0.21 [-0.47, 0.05] |
| --- | --- | --- | --- | --- |
| 1.44 Average Heart Rate [bpm] | 4 | 184 | Mean Difference (IV, Random, 95% CI [bpm]) | -2.45 [-5.61, 0.71] |

| 1.45 Average RPE | 3 | 97 | Mean Difference (IV, Random, 95% CI) | -0.15 [-1.04, 0.73] |
| --- | --- | --- | --- | --- |
| 1.46 POMS: Depression subscale | 2 | 41 | Std. Mean Difference (IV, Random, 95% CI) | 0.93 [0.15, 1.71] |

| 1.47 POMS: Anxiety subscale | 2 | 41 | Std. Mean Difference (IV, Random, 95% CI) | 0.68 [0.03, 1.32] |
| --- | --- | --- | --- | --- |
| 1.48 POMS: Vigour subscale | 2 | 41 | Std. Mean Difference (IV, Random, 95% CI) | -0.05 [-0.68, 0.57] |

| 1.49 Energy Intake (post-intervention) [kcal/day] | 5 | 236 | Mean Difference (IV, Random, 95% CI [kcal/day]) | 12.01 [-44.94, 68.95] |
| --- | --- | --- | --- | --- |
| 1.50 % Energy Intake from Fat (post-intervention) [%] | 3 | 186 | Mean Difference (IV, Random, 95% CI [%]) | -0.24 [-3.15, 2.67] |

**S3.2 Accumulated vs. Control**

| **Outcome or Subgroup** | **Studies** | **Participants** | **Statistical Method** | **Effect Estimate** |
| --- | --- | --- | --- | --- |

| 2.1 Maximal Oxygen Uptake | 4 | 223 | Std. Mean Difference (IV, Random, 95% CI) | 0.52 [0.24, 0.81] |
| --- | --- | --- | --- | --- |
| 2.2 Relative Maximal Oxygen Uptake [ml/kg/min] | 3 | 197 | Mean Difference (IV, Random, 95% CI [ml/kg/min]) | 2.32 [1.10, 3.54] |

| 2.3 Relative Maximal Oxygen Uptake (change) [ml/kg/min] | 2 | 110 | Mean Difference (IV, Random, 95% CI [ml/kg/min]) | 2.78 [2.51, 3.05] |
| --- | --- | --- | --- | --- |
| 2.4 Other cardiorespiratory fitness | 3 | 145 | Std. Mean Difference (IV, Random, 95% CI) | 0.24 [-0.18, 0.67] |

| 2.5 Resting Heart Rate (post-intervention) [bpm] | 1 | 40 | Mean Difference (IV, Random, 95% CI [bpm]) | -8.10 [-12.61, -3.59] |
| --- | --- | --- | --- | --- |
| 2.6 Systolic Blood Pressure (post-intervention) [mmHg] | 5 | 182 | Mean Difference (IV, Random, 95% CI [mmHg]) | -2.97 [-7.29, 1.34] |

| 2.7 Systolic Blood Pressure (change) [mmHg] | 2 | 45 | Mean Difference (IV, Random, 95% CI [mmHg]) | 1.34 [-12.65, 15.34] |
| --- | --- | --- | --- | --- |
| 2.8 Diastolic Blood Pressure (post-intervention) [mmHg] | 4 | 161 | Mean Difference (IV, Random, 95% CI [mmHg]) | -4.83 [-7.83, -1.84] |

| 2.9 Body mass (post-intervention) [Kg] | 4 | 164 | Mean Difference (IV, Random, 95% CI [Kg]) | -0.37 [-3.04, 2.30] |
| --- | --- | --- | --- | --- |
| 2.10 Body mass (change) [Kg] | 4 | 97 | Mean Difference (IV, Random, 95% CI [Kg]) | -1.94 [-3.42, -0.47] |

| 2.11 BMI (post-intervention) [kg/m2] | 3 | 147 | Mean Difference (IV, Random, 95% CI [kg/m2]) | -0.40 [-1.35, 0.55] |
| --- | --- | --- | --- | --- |
| 2.12 BMI (change) [kg/m2] | 2 | 48 | Mean Difference (IV, Random, 95% CI [kg/m2]) | -0.97 [-1.70, -0.24] |

| 2.13 Body Fat % (combined) [%] | 4 | 147 | Mean Difference (IV, Random, 95% CI [%]) | -0.92 [-1.78, -0.07] |
| --- | --- | --- | --- | --- |
| 2.14 Waist Circumference (post-intervention) [cm] | 3 | 77 | Mean Difference (IV, Random, 95% CI [cm]) | 1.79 [-7.56, 11.13] |

| 2.15 Waist Circumference (change) [cm] | 2 | 44 | Mean Difference (IV, Random, 95% CI [cm]) | -2.62 [-4.67, -0.56] |
| --- | --- | --- | --- | --- |
| 2.16 Hip Circumference (post-intervention) [cm] | 2 | 53 | Mean Difference (IV, Random, 95% CI [cm]) | 2.32 [-1.56, 6.19] |

| 2.17 Sum of skinfolds (change) [mm] | 3 | 70 | Mean Difference (IV, Random, 95% CI [mm]) | -6.39 [-8.25, -4.53] |
| --- | --- | --- | --- | --- |
| 2.18 Total Cholesterol (post-intervention) [mmol/L] | 3 | 127 | Mean Difference (IV, Random, 95% CI [mmol/L]) | 0.50 [-0.00, 1.00] |

| 2.19 LDL-Cholesterol (post-intervention) [mmol/L] | 3 | 127 | Mean Difference (IV, Random, 95% CI [mmol/L]) | 0.28 [-0.05, 0.62] |
| --- | --- | --- | --- | --- |
| 2.20 Triglycerides (post-intervention) [mmol/L] | 3 | 127 | Mean Difference (IV, Random, 95% CI [mmol/L]) | 0.03 [-0.13, 0.19] |

| 2.21 HDL-Cholesterol (post-intervention) [mmol/L] | 3 | 128 | Mean Difference (IV, Random, 95% CI [mmol/L]) | 0.14 [-0.16, 0.43] |
| --- | --- | --- | --- | --- |
| 2.22 Plasma Glucose (post-intervention) [mmol/L] | 2 | 111 | Mean Difference (IV, Random, 95% CI [mmol/L]) | -0.11 [-0.24, 0.02] |

| 2.23 Energy Intake (post-intervention) | 2 | 48 | Mean Difference (IV, Random, 95% CI) | -199.55 [-528.26, 129.16] |
| --- | --- | --- | --- | --- |

**S3.4 Subgroup analysis by exercise dose: Accumulated vs. Continuous Exercise**

| **Outcome or Subgroup** | **Studies** | **Participants** | **Statistical Method** | **Effect Estimate** |
| --- | --- | --- | --- | --- |

| 3.1 Maximal Oxygen Uptake (post-intervention) | 9 | 439 | Std. Mean Difference (IV, Random, 95% CI) | -0.00 [-0.19, 0.19] |
| --- | --- | --- | --- | --- |
| 3.1.1 <150 min | 2 | 144 | Std. Mean Difference (IV, Random, 95% CI) | -0.05 [-0.38, 0.28] |
| 3.1.2 150 min | 4 | 128 | Std. Mean Difference (IV, Random, 95% CI) | -0.02 [-0.41, 0.37] |
| 3.1.3 >150 min | 3 | 167 | Std. Mean Difference (IV, Random, 95% CI) | 0.08 [-0.33, 0.48] |
| 3.2 Relative Maximal Oxygen Uptake (post-intervention) [ml/kg/min] | 8 | 379 | Mean Difference (IV, Random, 95% CI [ml/kg/min]) | -0.50 [-1.53, 0.54] |
| 3.2.1 <150 min | 2 | 144 | Mean Difference (IV, Random, 95% CI [ml/kg/min]) | -0.30 [-2.02, 1.42] |
| 3.2.2 150 min | 4 | 116 | Mean Difference (IV, Random, 95% CI [ml/kg/min]) | -0.88 [-3.06, 1.30] |
| 3.2.3 >150 min | 2 | 119 | Mean Difference (IV, Random, 95% CI [ml/kg/min]) | -0.46 [-2.07, 1.16] |

| 3.3 Maximal Oxygen Uptake (change) | 6 | 288 | Std. Mean Difference (IV, Random, 95% CI) | -0.07 [-0.56, 0.42] |
| --- | --- | --- | --- | --- |
| 3.3.1 <150 min | 1 | 23 | Std. Mean Difference (IV, Random, 95% CI) | -0.08 [-0.90, 0.74] |
| 3.3.2 150 min | 3 | 102 | Std. Mean Difference (IV, Random, 95% CI) | 0.04 [-0.86, 0.93] |
| 3.3.3 >150 min | 2 | 163 | Std. Mean Difference (IV, Random, 95% CI) | -0.33 [-0.72, 0.06] |
| 3.4 Relative Maximal Oxygen Uptake (change) [ml/kg/min] | 4 | 125 | Mean Difference (IV, Random, 95% CI [ml/kg/min]) | 0.20 [-1.45, 1.86] |
| 3.4.1 <150 min | 1 | 23 | Mean Difference (IV, Random, 95% CI [ml/kg/min]) | -0.30 [-3.24, 2.64] |
| 3.4.2 150 min | 3 | 102 | Mean Difference (IV, Random, 95% CI [ml/kg/min]) | 0.31 [-1.78, 2.40] |
| 3.4.3 >150 min | 0 | 0 | Mean Difference (IV, Random, 95% CI [ml/kg/min]) | Not estimable |

| 3.5 Resting Heart Rate (post-intervention) [bpm] | 2 | 92 | Mean Difference (IV, Random, 95% CI [bpm]) | -2.54 [-5.83, 0.76] |
| --- | --- | --- | --- | --- |
| 3.5.1 <150 min | 1 | 52 | Mean Difference (IV, Random, 95% CI [bpm]) | -0.30 [-5.57, 4.97] |
| 3.5.2 150 min | 1 | 40 | Mean Difference (IV, Random, 95% CI [bpm]) | -3.80 [-7.64, 0.04] |
| 3.5.3 >150 min | 0 | 0 | Mean Difference (IV, Random, 95% CI [bpm]) | Not estimable |
| 3.6 Resting Heart Rate (change) [bpm] | 1 | 23 | Mean Difference (IV, Random, 95% CI [bpm]) | -4.30 [-18.42, 9.82] |
| 3.6.1 <150 min | 1 | 23 | Mean Difference (IV, Random, 95% CI [bpm]) | -4.30 [-18.42, 9.82] |

| 3.7 Systolic Blood Pressure (post-intervention) [mmHg] | 7 | 274 | Mean Difference (IV, Random, 95% CI [mmHg]) | -1.28 [-4.00, 1.43] |
| --- | --- | --- | --- | --- |
| 3.7.1 <150 min | 2 | 48 | Mean Difference (IV, Random, 95% CI [mmHg]) | -4.35 [-10.16, 1.46] |
| 3.7.2 150 min | 2 | 55 | Mean Difference (IV, Random, 95% CI [mmHg]) | -1.44 [-8.08, 5.19] |
| 3.7.3 >150 min | 3 | 171 | Mean Difference (IV, Random, 95% CI [mmHg]) | -0.15 [-3.62, 3.31] |
| 3.8 Systolic Blood Pressure (change) [mmHg] | 3 | 99 | Mean Difference (IV, Random, 95% CI [mmHg]) | 0.56 [-5.19, 6.31] |
| 3.8.1 <150 min | 1 | 23 | Mean Difference (IV, Random, 95% CI [mmHg]) | 8.90 [-0.04, 17.84] |
| 3.8.2 150 min | 1 | 24 | Mean Difference (IV, Random, 95% CI [mmHg]) | -2.80 [-8.11, 2.51] |
| 3.8.3 >150 min | 1 | 52 | Mean Difference (IV, Random, 95% CI [mmHg]) | -1.30 [-6.55, 3.95] |

| 3.9 Diastolic Blood Pressure (post-intervention) [mmHg] | 7 | 274 | Mean Difference (IV, Random, 95% CI [mmHg]) | -1.27 [-3.09, 0.56] |
| --- | --- | --- | --- | --- |
| 3.9.1 <150 min | 2 | 48 | Mean Difference (IV, Random, 95% CI [mmHg]) | -4.22 [-9.59, 1.16] |
| 3.9.2 150 min | 2 | 55 | Mean Difference (IV, Random, 95% CI [mmHg]) | -0.72 [-3.97, 2.53] |
| 3.9.3 >150 min | 3 | 171 | Mean Difference (IV, Random, 95% CI [mmHg]) | -0.33 [-2.60, 1.94] |
| 3.10 Diastolic Blood Pressure (change) [mmHg] | 2 | 75 | Mean Difference (IV, Random, 95% CI [mmHg]) | 3.35 [-1.59, 8.29] |
| 3.10.1 <150 min | 1 | 23 | Mean Difference (IV, Random, 95% CI [mmHg]) | 6.30 [0.74, 11.86] |
| 3.10.2 150 min | 0 | 0 | Mean Difference (IV, Random, 95% CI [mmHg]) | Not estimable |
| 3.10.3 >150 min | 1 | 52 | Mean Difference (IV, Random, 95% CI [mmHg]) | 1.20 [-2.71, 5.11] |

| 3.11 Body mass (post-intervention) [Kg] | 8 | 314 | Mean Difference (IV, Random, 95% CI [Kg]) | 0.59 [-1.77, 2.94] |
| --- | --- | --- | --- | --- |
| 3.11.1 <150 min | 2 | 48 | Mean Difference (IV, Random, 95% CI [Kg]) | -0.33 [-5.59, 4.93] |
| 3.11.2 150 min | 3 | 94 | Mean Difference (IV, Random, 95% CI [Kg]) | 2.93 [-3.84, 9.70] |
| 3.11.3 >150 min | 3 | 172 | Mean Difference (IV, Random, 95% CI [Kg]) | 0.44 [-2.43, 3.30] |
| 3.12 Body mass (change) [Kg] | 5 | 211 | Mean Difference (IV, Random, 95% CI [Kg]) | -0.92 [-1.59, -0.25] |
| 3.12.1 <150 min | 1 | 23 | Mean Difference (IV, Random, 95% CI [Kg]) | 0.01 [-1.73, 1.75] |
| 3.12.2 150 min | 2 | 54 | Mean Difference (IV, Random, 95% CI [Kg]) | -0.87 [-1.74, 0.01] |
| 3.12.3 >150 min | 2 | 134 | Mean Difference (IV, Random, 95% CI [Kg]) | -1.34 [-3.39, 0.72] |

| 3.13 BMI (post-intervention) [kg/m2] | 7 | 351 | Mean Difference (IV, Random, 95% CI [kg/m2]) | 0.12 [-0.46, 0.70] |
| --- | --- | --- | --- | --- |
| 3.13.1 <150 min | 2 | 44 | Mean Difference (IV, Random, 95% CI [kg/m2]) | 0.03 [-0.76, 0.82] |
| 3.13.2 150 min | 2 | 52 | Mean Difference (IV, Random, 95% CI [kg/m2]) | 2.08 [-0.75, 4.91] |
| 3.13.3 >150 min | 3 | 255 | Mean Difference (IV, Random, 95% CI [kg/m2]) | 0.04 [-0.85, 0.92] |
| 3.14 BMI (change) [kg/m2] | 3 | 72 | Mean Difference (IV, Random, 95% CI [kg/m2]) | -0.35 [-0.83, 0.13] |
| 3.14.1 <150 min | 1 | 23 | Mean Difference (IV, Random, 95% CI [kg/m2]) | 0.01 [-0.71, 0.73] |
| 3.14.2 150 min | 1 | 30 | Mean Difference (IV, Random, 95% CI [kg/m2]) | -0.20 [-0.67, 0.27] |
| 3.14.3 >150 min | 1 | 19 | Mean Difference (IV, Random, 95% CI [kg/m2]) | -0.85 [-1.46, -0.24] |

| 3.15 Body Fat (post-intervention) | 7 | 253 | Std. Mean Difference (IV, Random, 95% CI) | -0.07 [-0.33, 0.19] |
| --- | --- | --- | --- | --- |
| 3.15.1 <150 min | 3 | 67 | Std. Mean Difference (IV, Random, 95% CI) | 0.07 [-0.42, 0.55] |
| 3.15.2 150 min | 2 | 39 | Std. Mean Difference (IV, Random, 95% CI) | -0.13 [-0.77, 0.51] |
| 3.15.3 >150 min | 2 | 147 | Std. Mean Difference (IV, Random, 95% CI) | -0.12 [-0.46, 0.23] |
| 3.16 Body Fat % (combined) [%] | 7 | 251 | Mean Difference (IV, Random, 95% CI [%]) | -0.68 [-1.41, 0.05] |
| 3.16.1 <150 min | 3 | 67 | Mean Difference (IV, Random, 95% CI [%]) | 0.03 [-1.49, 1.55] |
| 3.16.2 150 min | 1 | 18 | Mean Difference (IV, Random, 95% CI [%]) | -2.10 [-9.18, 4.98] |
| 3.16.3 >150 min | 3 | 166 | Mean Difference (IV, Random, 95% CI [%]) | -0.87 [-1.71, -0.04] |

| 3.17 Waist Circumference (post-intervention) [cm] | 5 | 218 | Mean Difference (IV, Random, 95% CI [cm]) | 1.43 [-2.74, 5.60] |
| --- | --- | --- | --- | --- |
| 3.17.1 <150 min | 2 | 48 | Mean Difference (IV, Random, 95% CI [cm]) | 1.03 [-7.67, 9.72] |
| 3.17.2 150 min | 2 | 55 | Mean Difference (IV, Random, 95% CI [cm]) | 5.48 [-3.83, 14.79] |
| 3.17.3 >150 min | 1 | 115 | Mean Difference (IV, Random, 95% CI [cm]) | -0.30 [-5.65, 5.05] |
| 3.18 Waist Circumference (change) [cm] | 3 | 66 | Mean Difference (IV, Random, 95% CI [cm]) | -0.38 [-1.65, 0.88] |
| 3.18.1 <150 min | 1 | 23 | Mean Difference (IV, Random, 95% CI [cm]) | 0.88 [-1.49, 3.25] |
| 3.18.2 150 min | 1 | 24 | Mean Difference (IV, Random, 95% CI [cm]) | -1.20 [-3.12, 0.72] |
| 3.18.3 >150 min | 1 | 19 | Mean Difference (IV, Random, 95% CI [cm]) | -0.40 [-2.77, 1.97] |

| 3.19 Hip Circumference (post-intervention) [cm] | 3 | 72 | Mean Difference (IV, Random, 95% CI [cm]) | 2.32 [-2.65, 7.29] |
| --- | --- | --- | --- | --- |
| 3.19.1 <150 min | 1 | 17 | Mean Difference (IV, Random, 95% CI [cm]) | 1.30 [-6.26, 8.86] |
| 3.19.2 150 min | 2 | 55 | Mean Difference (IV, Random, 95% CI [cm]) | 3.23 [-3.94, 10.40] |
| 3.19.3 >150 min | 0 | 0 | Mean Difference (IV, Random, 95% CI [cm]) | Not estimable |
| 3.20 Waist-to-Hip Ratio (post-intervention) [cm] | 2 | 133 | Mean Difference (IV, Random, 95% CI [cm]) | 0.00 [-0.03, 0.03] |
| 3.20.1 <150 min | 0 | 0 | Mean Difference (IV, Random, 95% CI [cm]) | Not estimable |
| 3.20.2 150 min | 1 | 18 | Mean Difference (IV, Random, 95% CI [cm]) | -0.02 [-0.30, 0.26] |
| 3.20.3 >150 min | 1 | 115 | Mean Difference (IV, Random, 95% CI [cm]) | 0.01 [-0.03, 0.04] |

| 3.21 Sum of Skinfolds (change) [mm] | 3 | 69 | Mean Difference (IV, Random, 95% CI [mm]) | -0.15 [-1.88, 1.57] |
| --- | --- | --- | --- | --- |
| 3.21.1 <150 min | 0 | 0 | Mean Difference (IV, Random, 95% CI [mm]) | Not estimable |
| 3.21.2 150 min | 2 | 50 | Mean Difference (IV, Random, 95% CI [mm]) | -0.05 [-1.80, 1.70] |
| 3.21.3 >150 min | 1 | 19 | Mean Difference (IV, Random, 95% CI [mm]) | -4.10 [-14.83, 6.63] |
| 3.22 Total Cholesterol (post-intervention) [mmol/L] | 3 | 133 | Mean Difference (IV, Random, 95% CI [mmol/L]) | 0.13 [-0.21, 0.47] |
| 3.22.1 <150 min | 1 | 24 | Mean Difference (IV, Random, 95% CI [mmol/L]) | 0.00 [-0.80, 0.80] |
| 3.22.2 150 min | 1 | 21 | Mean Difference (IV, Random, 95% CI [mmol/L]) | -0.03 [-0.91, 0.85] |
| 3.22.3 >150 min | 1 | 88 | Mean Difference (IV, Random, 95% CI [mmol/L]) | 0.20 [-0.21, 0.61] |

| 3.23 Total Cholesterol (change) [mmol/L] | 2 | 41 | Mean Difference (IV, Random, 95% CI [mmol/L]) | -0.09 [-0.75, 0.57] |
| --- | --- | --- | --- | --- |
| 3.23.1 <150 min | 1 | 23 | Mean Difference (IV, Random, 95% CI [mmol/L]) | 0.09 [-0.71, 0.89] |
| 3.23.2 150 min | 1 | 18 | Mean Difference (IV, Random, 95% CI [mmol/L]) | -0.50 [-1.69, 0.69] |
| 3.23.3 >150 min | 0 | 0 | Mean Difference (IV, Random, 95% CI [mmol/L]) | Not estimable |
| 3.24 LDL-Cholesterol (post-intervention) [mmol/L] | 2 | 113 | Mean Difference (IV, Random, 95% CI [mmol/L]) | 0.08 [-0.27, 0.43] |
| 3.24.1 <150 min | 1 | 26 | Mean Difference (IV, Random, 95% CI [mmol/L]) | 0.00 [-0.79, 0.79] |
| 3.24.2 150 min | 0 | 0 | Mean Difference (IV, Random, 95% CI [mmol/L]) | Not estimable |
| 3.24.3 >150 min | 1 | 87 | Mean Difference (IV, Random, 95% CI [mmol/L]) | 0.10 [-0.29, 0.49] |

| 3.25 LDL-Cholesterol (change) [mmol/L] | 2 | 41 | Mean Difference (IV, Random, 95% CI [mmol/L]) | -0.39 [-0.73, -0.06] |
| --- | --- | --- | --- | --- |
| 3.25.1 <150 min | 1 | 23 | Mean Difference (IV, Random, 95% CI [mmol/L]) | 0.05 [-0.82, 0.92] |
| 3.25.2 150 min | 1 | 18 | Mean Difference (IV, Random, 95% CI [mmol/L]) | -0.46 [-0.59, -0.33] |
| 3.25.3 >150 min | 0 | 0 | Mean Difference (IV, Random, 95% CI [mmol/L]) | Not estimable |
| 3.26 Triglycerides (post-intervention) [mmol/L] | 3 | 134 | Mean Difference (IV, Random, 95% CI [mmol/L]) | 0.07 [-0.07, 0.21] |
| 3.26.1 <150 min | 1 | 26 | Mean Difference (IV, Random, 95% CI [mmol/L]) | 0.00 [-0.25, 0.25] |
| 3.26.2 150 min | 1 | 21 | Mean Difference (IV, Random, 95% CI [mmol/L]) | 0.03 [-0.55, 0.61] |
| 3.26.3 >150 min | 1 | 87 | Mean Difference (IV, Random, 95% CI [mmol/L]) | 0.11 [-0.06, 0.28] |

| 3.27 Triglycerides (change) [mmol/L] | 2 | 41 | Mean Difference (IV, Random, 95% CI [mmol/L]) | 0.00 [-0.06, 0.06] |
| --- | --- | --- | --- | --- |
| 3.27.1 <150 min | 1 | 23 | Mean Difference (IV, Random, 95% CI [mmol/L]) | 0.00 [-0.06, 0.06] |
| 3.27.2 150 min | 1 | 18 | Mean Difference (IV, Random, 95% CI [mmol/L]) | 0.72 [-0.92, 2.36] |
| 3.27.3 >150 min | 0 | 0 | Mean Difference (IV, Random, 95% CI [mmol/L]) | Not estimable |
| 3.28 HDL-Cholesterol (post-intervention) [mmol/L] | 3 | 135 | Mean Difference (IV, Random, 95% CI [mmol/L]) | -0.00 [-0.15, 0.15] |
| 3.28.1 <150 min | 1 | 26 | Mean Difference (IV, Random, 95% CI [mmol/L]) | -0.10 [-0.57, 0.37] |
| 3.28.2 150 min | 1 | 21 | Mean Difference (IV, Random, 95% CI [mmol/L]) | -0.06 [-0.46, 0.34] |
| 3.28.3 >150 min | 1 | 88 | Mean Difference (IV, Random, 95% CI [mmol/L]) | 0.02 [-0.15, 0.19] |

| 3.29 HDL-Cholesterol (change) [mmol/L] | 2 | 41 | Mean Difference (IV, Random, 95% CI [mmol/L]) | 0.00 [-0.05, 0.05] |
| --- | --- | --- | --- | --- |
| 3.29.1 <150 min | 1 | 23 | Mean Difference (IV, Random, 95% CI [mmol/L]) | 0.00 [-0.05, 0.05] |
| 3.29.2 150 min | 1 | 18 | Mean Difference (IV, Random, 95% CI [mmol/L]) | 0.10 [-0.26, 0.46] |
| 3.29.3 >150 min | 0 | 0 | Mean Difference (IV, Random, 95% CI [mmol/L]) | Not estimable |
| 3.30 Fasting Blood Glucose (combined) [mmol/L] | 2 | 112 | Mean Difference (IV, Random, 95% CI [mmol/L]) | -0.02 [-0.23, 0.20] |
| 3.30.1 <150 min | 1 | 23 | Mean Difference (IV, Random, 95% CI [mmol/L]) | -0.12 [-0.17, -0.07] |
| 3.30.2 150 min | 0 | 0 | Mean Difference (IV, Random, 95% CI [mmol/L]) | Not estimable |
| 3.30.3 >150 min | 1 | 89 | Mean Difference (IV, Random, 95% CI [mmol/L]) | 0.10 [-0.04, 0.24] |

| 3.31 Fasting Insulin (combined) [mlU/L] | 2 | 112 | Mean Difference (IV, Random, 95% CI [mlU/L]) | 0.37 [-0.32, 1.06] |
| --- | --- | --- | --- | --- |
| 3.31.1 <150 min | 1 | 23 | Mean Difference (IV, Random, 95% CI [mlU/L]) | -0.06 [-1.25, 1.14] |
| 3.31.2 150 min | 0 | 0 | Mean Difference (IV, Random, 95% CI [mlU/L]) | Not estimable |
| 3.31.3 >150 min | 1 | 89 | Mean Difference (IV, Random, 95% CI [mlU/L]) | 0.58 [-0.26, 1.42] |
| 3.32 Total Minutes of Physical Activity Completed [minutes] | 3 | 84 | Mean Difference (IV, Random, 95% CI [minutes]) | -20.31 [-93.97, 53.36] |
| 3.32.1 <150 min | 2 | 60 | Mean Difference (IV, Random, 95% CI [minutes]) | -24.98 [-153.13, 103.17] |
| 3.32.2 150 min | 1 | 24 | Mean Difference (IV, Random, 95% CI [minutes]) | -18.00 [-108.03, 72.03] |
| 3.32.3 >150 min | 0 | 0 | Mean Difference (IV, Random, 95% CI [minutes]) | Not estimable |

| 3.33 Average days/week of Physical Activity [days/week] | 3 | 80 | Mean Difference (IV, Random, 95% CI [days/week]) | -0.26 [-0.59, 0.07] |
| --- | --- | --- | --- | --- |
| 3.33.1 <150 min | 0 | 0 | Mean Difference (IV, Random, 95% CI [days/week]) | Not estimable |
| 3.33.2 150 min | 1 | 32 | Mean Difference (IV, Random, 95% CI [days/week]) | -0.20 [-0.56, 0.16] |
| 3.33.3 >150 min | 2 | 48 | Mean Difference (IV, Random, 95% CI [days/week]) | -0.56 [-1.37, 0.24] |
| 3.34 % Completed of Prescribed Sessions | 11 | 601 | Mean Difference (IV, Random, 95% CI) | -3.88 [-6.92, -0.84] |
| 3.34.1 <150 min | 4 | 191 | Mean Difference (IV, Random, 95% CI) | -4.66 [-12.47, 3.14] |
| 3.34.2 150 min | 3 | 85 | Mean Difference (IV, Random, 95% CI) | -3.07 [-4.47, -1.68] |
| 3.34.3 >150 min | 4 | 325 | Mean Difference (IV, Random, 95% CI) | -10.97 [-29.41, 7.47] |

| 3.35 Self-reported Average Physical Activity | 7 | 363 | Std. Mean Difference (IV, Random, 95% CI) | 0.29 [-0.08, 0.66] |
| --- | --- | --- | --- | --- |
| 3.35.1 <150 min | 1 | 37 | Std. Mean Difference (IV, Random, 95% CI) | -0.02 [-0.66, 0.63] |
| 3.35.2 150 min | 1 | 21 | Std. Mean Difference (IV, Random, 95% CI) | 1.95 [0.85, 3.05] |
| 3.35.3 >150 min | 5 | 305 | Std. Mean Difference (IV, Random, 95% CI) | 0.22 [-0.06, 0.50] |
| 3.36 Self-reported Physical Activity Min/week [min/week] | 5 | 244 | Mean Difference (IV, Random, 95% CI [min/week]) | 6.85 [-4.52, 18.22] |
| 3.36.1 <150 min | 1 | 37 | Mean Difference (IV, Random, 95% CI [min/week]) | -0.30 [-11.93, 11.33] |
| 3.36.2 150 min | 1 | 21 | Mean Difference (IV, Random, 95% CI [min/week]) | 11.00 [5.22, 16.78] |
| 3.36.3 >150 min | 3 | 186 | Mean Difference (IV, Random, 95% CI [min/week]) | 15.77 [-14.67, 46.21] |

| 3.37 Self-reported Average Physical Activity [min/day] | 3 | 138 | Mean Difference (IV, Random, 95% CI [min/day]) | -0.51 [-4.77, 3.75] |
| --- | --- | --- | --- | --- |
| 3.37.1 <150 min | 0 | 0 | Mean Difference (IV, Random, 95% CI [min/day]) | Not estimable |
| 3.37.2 150 min | 0 | 0 | Mean Difference (IV, Random, 95% CI [min/day]) | Not estimable |
| 3.37.3 >150 min | 3 | 138 | Mean Difference (IV, Random, 95% CI [min/day]) | -0.51 [-4.77, 3.75] |
| 3.38 Objective Average Physical Activity | 6 | 523 | Std. Mean Difference (IV, Random, 95% CI) | 0.25 [0.01, 0.49] |
| 3.38.1 <150 min | 0 | 0 | Std. Mean Difference (IV, Random, 95% CI) | Not estimable |
| 3.38.2 150 min | 2 | 103 | Std. Mean Difference (IV, Random, 95% CI) | 0.04 [-0.38, 0.45] |
| 3.38.3 >150 min | 4 | 420 | Std. Mean Difference (IV, Random, 95% CI) | 0.33 [0.04, 0.61] |

| 3.39 Pedometer measured Physical Activity | 4 | 219 | Std. Mean Difference (IV, Random, 95% CI) | -0.00 [-0.44, 0.43] |
| --- | --- | --- | --- | --- |
| 3.39.1 <150 min | 0 | 0 | Std. Mean Difference (IV, Random, 95% CI) | Not estimable |
| 3.39.2 150 min | 2 | 103 | Std. Mean Difference (IV, Random, 95% CI) | 0.04 [-0.38, 0.45] |
| 3.39.3 >150 min | 2 | 116 | Std. Mean Difference (IV, Random, 95% CI) | -0.12 [-1.22, 0.97] |
| 3.40 Heart Rate | 6 | 227 | Std. Mean Difference (IV, Random, 95% CI) | -0.21 [-0.47, 0.05] |
| 3.40.1 <150 min | 2 | 61 | Std. Mean Difference (IV, Random, 95% CI) | 0.05 [-0.46, 0.55] |
| 3.40.2 150 min | 2 | 60 | Std. Mean Difference (IV, Random, 95% CI) | -0.36 [-0.87, 0.15] |
| 3.40.3 >150 min | 2 | 106 | Std. Mean Difference (IV, Random, 95% CI) | -0.26 [-0.65, 0.12] |

| 3.41 Average Heart Rate [bpm] | 4 | 184 | Mean Difference (IV, Random, 95% CI [bpm]) | -2.45 [-5.61, 0.71] |
| --- | --- | --- | --- | --- |
| 3.41.1 <150 min | 1 | 37 | Mean Difference (IV, Random, 95% CI [bpm]) | 0.00 [-7.76, 7.76] |
| 3.41.2 150 min | 2 | 60 | Mean Difference (IV, Random, 95% CI [bpm]) | -4.16 [-9.41, 1.09] |
| 3.41.3 >150 min | 1 | 87 | Mean Difference (IV, Random, 95% CI [bpm]) | -2.00 [-6.60, 2.60] |
| 3.42 Average RPE | 3 | 97 | Mean Difference (IV, Random, 95% CI) | -0.15 [-1.04, 0.73] |
| 3.42.1 <150 min | 2 | 61 | Mean Difference (IV, Random, 95% CI) | 0.24 [-0.45, 0.92] |
| 3.42.2 150 min | 1 | 36 | Mean Difference (IV, Random, 95% CI) | -1.00 [-1.98, -0.02] |
| 3.42.3 >150 min | 0 | 0 | Mean Difference (IV, Random, 95% CI) | Not estimable |

| 3.43 Energy Intake (post-intervention) [kcal/day] | 4 | 216 | Mean Difference (IV, Random, 95% CI [kcal/day]) | 18.82 [-39.77, 77.42] |
| --- | --- | --- | --- | --- |
| 3.43.1 <150 min | 0 | 0 | Mean Difference (IV, Random, 95% CI [kcal/day]) | Not estimable |
| 3.43.2 150 min | 1 | 30 | Mean Difference (IV, Random, 95% CI [kcal/day]) | 14.00 [-48.32, 76.32] |
| 3.43.3 >150 min | 3 | 186 | Mean Difference (IV, Random, 95% CI [kcal/day]) | 55.63 [-116.55, 227.80] |
| 3.44 % Energy Intake from Fat (post-intervention) [%] | 3 | 186 | Mean Difference (IV, Random, 95% CI [%]) | -0.24 [-3.15, 2.67] |
| 3.44.1 <150 min | 0 | 0 | Mean Difference (IV, Random, 95% CI [%]) | Not estimable |
| 3.44.2 150 min | 0 | 0 | Mean Difference (IV, Random, 95% CI [%]) | Not estimable |
| 3.44.3 >150 min | 3 | 186 | Mean Difference (IV, Random, 95% CI [%]) | -0.24 [-3.15, 2.67] |

**S3.Top of Form**

**Bottom of Form**

**5 Subgroup analysis by exercise dose: Accumulated vs. Control**

| **Outcome or Subgroup** | **Studies** | **Participants** | **Statistical Method** | **Effect Estimate** |
| --- | --- | --- | --- | --- |

| 4.1 Maximal Oxygen Uptake | 4 | 223 | Std. Mean Difference (IV, Random, 95% CI) | 0.52 [0.24, 0.81] |
| --- | --- | --- | --- | --- |
| 4.1.1 <150 min | 1 | 92 | Std. Mean Difference (IV, Random, 95% CI) | 0.28 [-0.14, 0.70] |
| 4.1.2 150 min | 2 | 45 | Std. Mean Difference (IV, Random, 95% CI) | 0.47 [-0.15, 1.09] |
| 4.1.3 >150 min | 1 | 86 | Std. Mean Difference (IV, Random, 95% CI) | 0.82 [0.38, 1.26] |
| 4.2 Relative Maximal Oxygen Uptake [ml/kg/min] | 3 | 197 | Mean Difference (IV, Random, 95% CI [ml/kg/min]) | 2.32 [1.10, 3.54] |
| 4.2.1 <150 min | 1 | 92 | Mean Difference (IV, Random, 95% CI [ml/kg/min]) | 1.30 [-0.58, 3.18] |
| 4.2.2 150 min | 1 | 19 | Mean Difference (IV, Random, 95% CI [ml/kg/min]) | 1.96 [-3.00, 6.92] |
| 4.2.3 >150 min | 1 | 86 | Mean Difference (IV, Random, 95% CI [ml/kg/min]) | 3.10 [1.51, 4.69] |

| 4.3 Relative Maximal Oxygen Uptake (change) [ml/kg/min] | 2 | 110 | Mean Difference (IV, Random, 95% CI [ml/kg/min]) | 2.78 [2.51, 3.05] |
| --- | --- | --- | --- | --- |
| 4.3.1 <150 min | 0 | 0 | Mean Difference (IV, Random, 95% CI [ml/kg/min]) | Not estimable |
| 4.3.2 150 min | 1 | 24 | Mean Difference (IV, Random, 95% CI [ml/kg/min]) | 2.80 [2.52, 3.08] |
| 4.3.3 >150 min | 1 | 86 | Mean Difference (IV, Random, 95% CI [ml/kg/min]) | 2.50 [1.50, 3.50] |
| 4.4 Resting Heart Rate (post-intervention) [bpm] | 1 | 40 | Mean Difference (IV, Random, 95% CI [bpm]) | -8.10 [-12.61, -3.59] |
| 4.4.1 <150 min | 0 | 0 | Mean Difference (IV, Random, 95% CI [bpm]) | Not estimable |
| 4.4.2 150 min | 1 | 40 | Mean Difference (IV, Random, 95% CI [bpm]) | -8.10 [-12.61, -3.59] |
| 4.4.3 >150 min | 0 | 0 | Mean Difference (IV, Random, 95% CI [bpm]) | Not estimable |

| 4.5 Systolic Blood Pressure (post-intervention) [mmHg] | 5 | 182 | Mean Difference (IV, Random, 95% CI [mmHg]) | -2.97 [-7.29, 1.34] |
| --- | --- | --- | --- | --- |
| 4.5.1 <150 min | 2 | 41 | Mean Difference (IV, Random, 95% CI [mmHg]) | -3.67 [-12.87, 5.53] |
| 4.5.2 150 min | 2 | 55 | Mean Difference (IV, Random, 95% CI [mmHg]) | -5.09 [-12.47, 2.29] |
| 4.5.3 >150 min | 1 | 86 | Mean Difference (IV, Random, 95% CI [mmHg]) | 0.90 [-5.58, 7.38] |
| 4.6 Systolic Blood Pressure (change) [mmHg] | 2 | 45 | Mean Difference (IV, Random, 95% CI [mmHg]) | 1.34 [-12.65, 15.34] |
| 4.6.1 <150 min | 1 | 23 | Mean Difference (IV, Random, 95% CI [mmHg]) | 8.90 [-0.04, 17.84] |
| 4.6.2 150 min | 1 | 22 | Mean Difference (IV, Random, 95% CI [mmHg]) | -5.40 [-11.35, 0.55] |
| 4.6.3 >150 min | 0 | 0 | Mean Difference (IV, Random, 95% CI [mmHg]) | Not estimable |

| 4.7 Diastolic Blood Pressure (post-intervention) [mmHg] | 4 | 161 | Mean Difference (IV, Random, 95% CI [mmHg]) | -4.83 [-7.83, -1.84] |
| --- | --- | --- | --- | --- |
| 4.7.1 <150 min | 2 | 41 | Mean Difference (IV, Random, 95% CI [mmHg]) | -2.74 [-12.94, 7.46] |
| 4.7.2 150 min | 1 | 34 | Mean Difference (IV, Random, 95% CI [mmHg]) | -5.80 [-9.81, -1.79] |
| 4.7.3 >150 min | 1 | 86 | Mean Difference (IV, Random, 95% CI [mmHg]) | -4.70 [-8.77, -0.63] |
| 4.8 Body mass (post-intervention) [Kg] | 4 | 164 | Mean Difference (IV, Random, 95% CI [Kg]) | -0.37 [-3.04, 2.30] |
| 4.8.1 <150 min | 2 | 41 | Mean Difference (IV, Random, 95% CI [Kg]) | -1.09 [-5.43, 3.25] |
| 4.8.2 150 min | 1 | 36 | Mean Difference (IV, Random, 95% CI [Kg]) | 5.50 [-14.01, 25.01] |
| 4.8.3 >150 min | 1 | 87 | Mean Difference (IV, Random, 95% CI [Kg]) | -0.10 [-3.55, 3.35] |

| 4.9 Body mass (change) [Kg] | 4 | 97 | Mean Difference (IV, Random, 95% CI [Kg]) | -1.94 [-3.42, -0.47] |
| --- | --- | --- | --- | --- |
| 4.9.1 <150 min | 1 | 23 | Mean Difference (IV, Random, 95% CI [Kg]) | 0.01 [-1.73, 1.75] |
| 4.9.2 150 min | 2 | 52 | Mean Difference (IV, Random, 95% CI [Kg]) | -3.01 [-4.34, -1.68] |
| 4.9.3 >150 min | 1 | 22 | Mean Difference (IV, Random, 95% CI [Kg]) | -1.30 [-2.82, 0.22] |
| 4.10 BMI (post-intervention) [kg/m2] | 3 | 147 | Mean Difference (IV, Random, 95% CI [kg/m2]) | -0.40 [-1.35, 0.55] |
| 4.10.1 <150 min | 1 | 24 | Mean Difference (IV, Random, 95% CI [kg/m2]) | -1.16 [-2.89, 0.57] |
| 4.10.2 150 min | 1 | 36 | Mean Difference (IV, Random, 95% CI [kg/m2]) | 1.10 [-5.54, 7.74] |
| 4.10.3 >150 min | 1 | 87 | Mean Difference (IV, Random, 95% CI [kg/m2]) | -0.10 [-1.26, 1.06] |

| 4.11 BMI (change) [kg/m2] | 2 | 48 | Mean Difference (IV, Random, 95% CI [kg/m2]) | -0.97 [-1.70, -0.24] |
| --- | --- | --- | --- | --- |
| 4.11.1 <150 min | 0 | 0 | Mean Difference (IV, Random, 95% CI [kg/m2]) | Not estimable |
| 4.11.2 150 min | 1 | 26 | Mean Difference (IV, Random, 95% CI [kg/m2]) | -1.30 [-1.63, -0.97] |
| 4.11.3 >150 min | 1 | 22 | Mean Difference (IV, Random, 95% CI [kg/m2]) | -0.55 [-1.15, 0.05] |
| 4.12 Body Fat % (combined) [%] | 3 | 83 | Mean Difference (IV, Random, 95% CI [%]) | -0.75 [-1.46, -0.04] |
| 4.12.1 <150 min | 2 | 39 | Mean Difference (IV, Random, 95% CI [%]) | -1.39 [-4.75, 1.96] |
| 4.12.2 150 min | 0 | 0 | Mean Difference (IV, Random, 95% CI [%]) | Not estimable |
| 4.12.3 >150 min | 1 | 44 | Mean Difference (IV, Random, 95% CI [%]) | -0.72 [-1.44, 0.00] |

| 4.13 Waist Circumference (post-intervention) [cm] | 3 | 77 | Mean Difference (IV, Random, 95% CI [cm]) | 1.79 [-7.56, 11.13] |
| --- | --- | --- | --- | --- |
| 4.13.1 <150 min | 2 | 41 | Mean Difference (IV, Random, 95% CI [cm]) | -0.06 [-11.32, 11.20] |
| 4.13.2 150 min | 1 | 36 | Mean Difference (IV, Random, 95% CI [cm]) | 7.60 [-5.33, 20.53] |
| 4.13.3 >150 min | 0 | 0 | Mean Difference (IV, Random, 95% CI [cm]) | Not estimable |
| 4.14 Waist Circumference (change) [cm] | 2 | 44 | Mean Difference (IV, Random, 95% CI [cm]) | -2.62 [-4.67, -0.56] |
| 4.14.1 <150 min | 0 | 0 | Mean Difference (IV, Random, 95% CI [cm]) | Not estimable |
| 4.14.2 150 min | 1 | 22 | Mean Difference (IV, Random, 95% CI [cm]) | -3.60 [-5.09, -2.11] |
| 4.14.3 >150 min | 1 | 22 | Mean Difference (IV, Random, 95% CI [cm]) | -1.50 [-3.32, 0.32] |

| 4.15 Hip Circumference (post-intervention) [cm] | 2 | 53 | Mean Difference (IV, Random, 95% CI [cm]) | 2.32 [-1.56, 6.19] |
| --- | --- | --- | --- | --- |
| 4.15.1 <150 min | 1 | 17 | Mean Difference (IV, Random, 95% CI [cm]) | 1.90 [-2.15, 5.95] |
| 4.15.2 150 min | 1 | 36 | Mean Difference (IV, Random, 95% CI [cm]) | 6.80 [-6.47, 20.07] |
| 4.15.3 >150 min | 0 | 0 | Mean Difference (IV, Random, 95% CI [cm]) | Not estimable |
| 4.16 Sum of skinfolds (change) [mm] | 3 | 70 | Mean Difference (IV, Random, 95% CI [mm]) | -6.39 [-8.25, -4.53] |
| 4.16.1 <150 min | 0 | 0 | Mean Difference (IV, Random, 95% CI [mm]) | Not estimable |
| 4.16.2 150 min | 2 | 48 | Mean Difference (IV, Random, 95% CI [mm]) | -6.46 [-8.38, -4.54] |
| 4.16.3 >150 min | 1 | 22 | Mean Difference (IV, Random, 95% CI [mm]) | -5.30 [-12.80, 2.20] |

| 4.17 Total Cholesterol (post-intervention) [mmol/L] | 2 | 103 | Mean Difference (IV, Random, 95% CI [mmol/L]) | 0.44 [-0.17, 1.05] |
| --- | --- | --- | --- | --- |
| 4.17.1 <150 min | 1 | 17 | Mean Difference (IV, Random, 95% CI [mmol/L]) | 0.80 [0.17, 1.43] |
| 4.17.2 150 min | 0 | 0 | Mean Difference (IV, Random, 95% CI [mmol/L]) | Not estimable |
| 4.17.3 >150 min | 1 | 86 | Mean Difference (IV, Random, 95% CI [mmol/L]) | 0.17 [-0.23, 0.57] |
| 4.18 LDL-Cholesterol (post-intervention) [mmol/L] | 2 | 103 | Mean Difference (IV, Random, 95% CI [mmol/L]) | 0.34 [-0.20, 0.87] |
| 4.18.1 <150 min | 1 | 18 | Mean Difference (IV, Random, 95% CI [mmol/L]) | 0.70 [-0.01, 1.41] |
| 4.18.2 150 min | 0 | 0 | Mean Difference (IV, Random, 95% CI [mmol/L]) | Not estimable |
| 4.18.3 >150 min | 1 | 85 | Mean Difference (IV, Random, 95% CI [mmol/L]) | 0.13 [-0.27, 0.53] |

| 4.19 Triglycerides (post-intervention) [mmol/L] | 2 | 103 | Mean Difference (IV, Random, 95% CI [mmol/L]) | 0.05 [-0.15, 0.25] |
| --- | --- | --- | --- | --- |
| 4.19.1 <150 min | 1 | 18 | Mean Difference (IV, Random, 95% CI [mmol/L]) | 0.20 [-0.12, 0.52] |
| 4.19.2 150 min | 0 | 0 | Mean Difference (IV, Random, 95% CI [mmol/L]) | Not estimable |
| 4.19.3 >150 min | 1 | 85 | Mean Difference (IV, Random, 95% CI [mmol/L]) | -0.02 [-0.21, 0.17] |
| 4.20 HDL-Cholesterol (post-intervention) [mmol/L] | 2 | 104 | Mean Difference (IV, Random, 95% CI [mmol/L]) | 0.01 [-0.15, 0.17] |
| 4.20.1 <150 min | 1 | 18 | Mean Difference (IV, Random, 95% CI [mmol/L]) | 0.00 [-0.51, 0.51] |
| 4.20.2 150 min | 0 | 0 | Mean Difference (IV, Random, 95% CI [mmol/L]) | Not estimable |
| 4.20.3 >150 min | 1 | 86 | Mean Difference (IV, Random, 95% CI [mmol/L]) | 0.01 [-0.16, 0.18] |

| 4.21 Energy Intake (post-intervention) | 2 | 48 | Mean Difference (IV, Random, 95% CI) | -199.55 [-528.26, 129.16] |
| --- | --- | --- | --- | --- |
| 4.21.1 <150 min | 0 | 0 | Mean Difference (IV, Random, 95% CI) | Not estimable |
| 4.21.2 150 min | 1 | 26 | Mean Difference (IV, Random, 95% CI) | 16.40 [-456.70, 489.50] |
| 4.21.3 >150 min | 1 | 22 | Mean Difference (IV, Random, 95% CI) | -329.80 [-661.23, 1.63] |
